# Immediate-Early, Early, and Late Responses to DNA Double Stranded Breaks

**DOI:** 10.3389/fgene.2022.793884

**Published:** 2022-01-31

**Authors:** Shaylee R. Kieffer, Noel F. Lowndes

**Affiliations:** Centre for Chromosome Biology (CCB), Biomedical Sciences Building (BSB), School of Biological and Chemical Sciences, National University of Ireland, Galway (NUIG), Galway, Ireland

**Keywords:** DNA repair, double strand breaks (DSBs), Immediate-early response, Early response, Late response, pre-repair responses, non-homologous end joining (NHEJ), homologous recombination (HR)

## Abstract

Loss or rearrangement of genetic information can result from incorrect responses to DNA double strand breaks (DSBs). The cellular responses to DSBs encompass a range of highly coordinated events designed to detect and respond appropriately to the damage, thereby preserving genomic integrity. In analogy with events occurring during viral infection, we appropriate the terms Immediate-Early, Early, and Late to describe the pre-repair responses to DSBs. A distinguishing feature of the Immediate-Early response is that the large protein condensates that form during the Early and Late response and are resolved upon repair, termed foci, are not visible. The Immediate-Early response encompasses initial lesion sensing, involving poly (ADP-ribose) polymerases (PARPs), KU70/80, and MRN, as well as rapid repair by so-called ‘fast-kinetic’ canonical non-homologous end joining (cNHEJ). Initial binding of PARPs and the KU70/80 complex to breaks appears to be mutually exclusive at easily ligatable DSBs that are repaired efficiently by fast-kinetic cNHEJ; a process that is PARP-, ATM-, 53BP1-, Artemis-, and resection-independent. However, at more complex breaks requiring processing, the Immediate-Early response involving PARPs and the ensuing highly dynamic PARylation (polyADP ribosylation) of many substrates may aid recruitment of both KU70/80 and MRN to DSBs. Complex DSBs rely upon the Early response, largely defined by ATM-dependent focal recruitment of many signalling molecules into large condensates, and regulated by complex chromatin dynamics. Finally, the Late response integrates information from cell cycle phase, chromatin context, and type of DSB to determine appropriate pathway choice. Critical to pathway choice is the recruitment of p53 binding protein 1 (53BP1) and breast cancer associated 1 (BRCA1). However, additional factors recruited throughout the DSB response also impact upon pathway choice, although these remain to be fully characterised. The Late response somehow channels DSBs into the appropriate high-fidelity repair pathway, typically either ‘slow-kinetic’ cNHEJ or homologous recombination (HR). Loss of specific components of the DSB repair machinery results in cells utilising remaining factors to effect repair, but often at the cost of increased mutagenesis. Here we discuss the complex regulation of the Immediate-Early, Early, and Late responses to DSBs proceeding repair itself.

## Introduction to DNA Damage Responses

**FIGURE 1:**
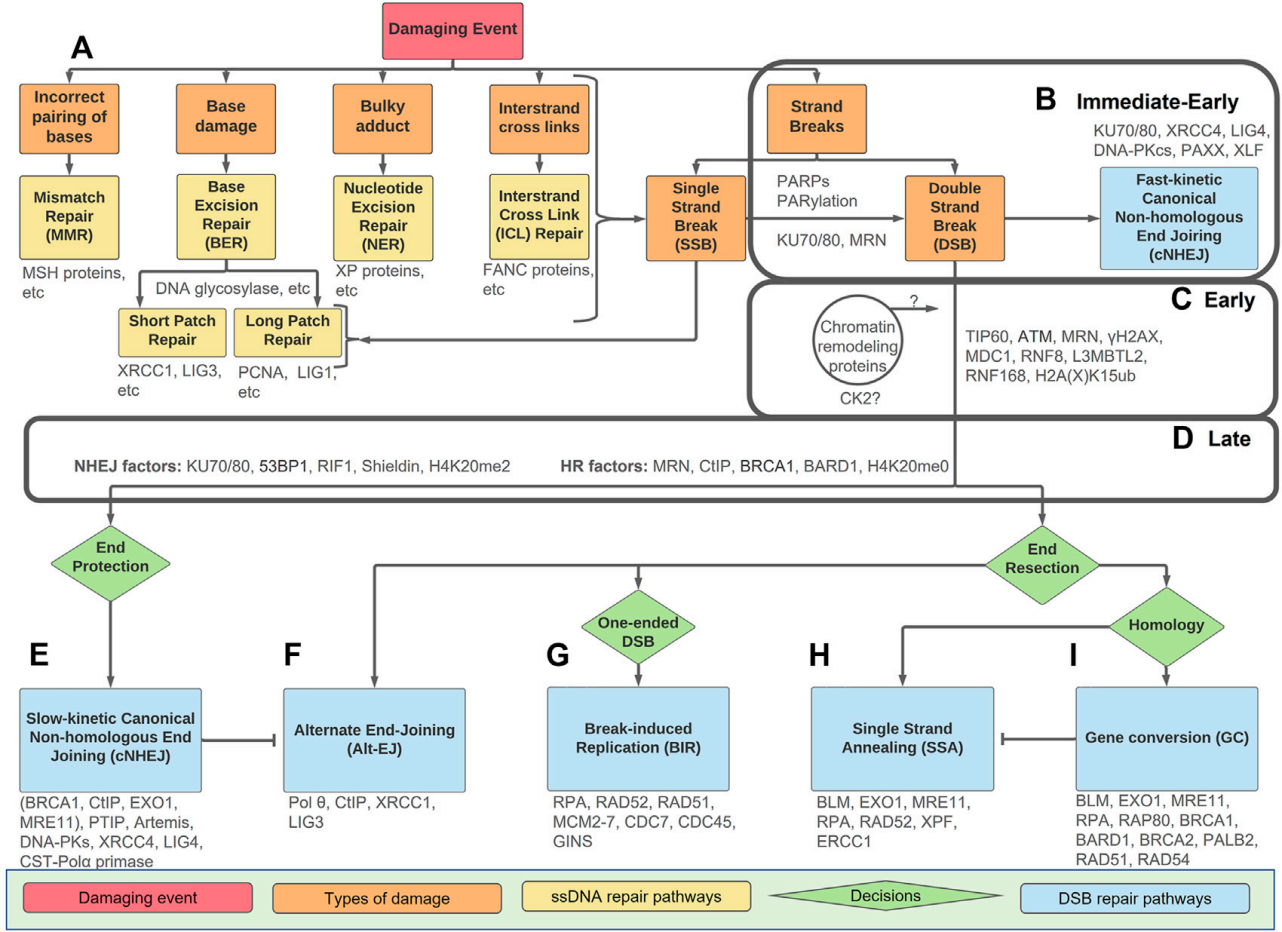
An overview of types of DNA damage and repair pathways with the Immediate-Early, Early, and Late stage indicated. The Immediate-Early, Early, and Late stages of the DSB response are indicated in the boxes. **(A)** Mismatched nucleotides or DNA damage (orange boxes) can be divided into five categories and directed towards the repair pathways as indicated (yellow). Any form of damage can become SSBs or DSBs if not repaired correctly. **(B)** Strand breaks, either SSBs or DSB, have a PARP/PARylation response. In the Immediate-Early response, the PARP/PARylation can facilitate recruitment of KU70/80 and MRN to some DSBs. If the break is easily ligatable, fast-kinetic cNHEJ (blue) repairs the damage within the Immediate-Early response without any requirement for PARylation, processing or ATM-dependent signalling. **(C)** If the break requires processing prior to repair, the Early response is activated. This includes ATM-dependent signalling which requires dynamic chromatin remodelling. The Early response culminates in the ubiquitination of H2A(X)K15. How chromatin events and CK2 activation tie into these processes remains unclear. **(D)** The Late response includes 53BP1 and BRCA1-BARD1 as ‘readers’ of the H2A(X) ubiquitin mark as well as the methylation state of H4K20. The Late response occurs prior to pathway choice and includes an intricate balance of end-resection vs. end-protection machinery. **(E–I)** Downstream repair pathways (blue) with decision points between pathways (green). Slow-kinetic cNHEJ and GC are high-fidelity repairs, while Alt-EJ, BIR, and SSA, are mutagenic and result when repair machinery is not available. Key proteins discussed are in grey.

The DNA damage response (DDR) encompasses a network of biological pathways that detect and respond to various forms of DNA damage using a multitude of distinct and overlapping cellular mechanisms (Figure 1 and Lindahl and Barnes, 2000; Jackson, 2002). As an estimated 10^5^ lesions occur per cell per day in human cells, proper coordination of the DDR is essential to preserving genomic integrity (Lindahl, 1993; Hoeijmakers, 2009). Repair, cell cycle arrest, senescence, and apoptosis all represent biological responses to DNA damage that are dependent upon cell type and severity of damage (Jackson and Bartek, 2009). Correct coordination of the DDR protects the genome from the accumulation of mutations, ranging from simple nucleotide changes to complex chromosomal alterations such as those generated during chromothripsis (Jackson, 2002; Jackson and Bartek, 2009; Forment et al., 2012).

Interestingly, endogenous sources of DNA damage due to normal metabolism (e.g., errors during DNA metabolism or chemical attack by indigenous metabolites), rather than exogenous sources (e.g., radiation and environmental chemicals), are more important with respect to generating mutations that drive the cancerous phenotype (Tomasetti and Vogelstein, 2015). DNA damage comes in many forms, including incorrect hydrogen-bonding, bulky adducts, base damage, intrastrand cross-links, and damage to the sugar phosphate backbone (Figure 1A and Lindahl, 1993; Lindahl and Barnes, 2000; Jackson and Bartek, 2009). Incorrect pairing of bases is corrected by mismatch repair (MMR), bulky adducts by nucleotide excision repair (NER), and base damage by base excision repair (BER) (Friedberg, 2001; Jiricny, 2006; Krokan and Bjørås, 2013). The two strands of DNA can also be chemically cross-linked together and resolved by interstrand crosslink (ICL) repair, a highly complex pathway utilising proteins involved in other DNA repair pathways, as well as others identified as deficient in Fanconi anaemia (FA) (Deans and West, 2011; Semlow and Walter, 2021). Interestingly, in addition to having a dedicated FA core complex regulating E3 ubiquitin ligase activity, many of the other associated FANC proteins overlap with downstream double strand break (DSB) repair proteins, as a DSB forms transiently during the unhooking step required for repair of the crosslinks. These proteins include BRCA1, BRCA2, PALB2, RAD51, XRCC2, XPF, and REV7. Any of the lesions to the nucleotide, be it a simple base damage or a more complex event, can become strand breaks within the sugar-phosphate backbone if not repaired correctly (Figure 1A).

Single strand break (SSB) repair is often considered a ‘specialised’ form of BER, as most SSB repair proteins are also involved in either short-patch or long-patch repair, despite the damage being to the sugar-phosphate backbone instead of the base (Caldecott, 2014a). In BER, distinct DNA glycosylases recognise specific types of base damage, excise the damaged base, cleave the resulting abasic site, fill in the single nucleotide gap (or multiple nucleotides in long-patch BER), and ligate the nick (Krokan and Bjørås, 2013). If the ligation step does not occur, or if the damage escapes detection prior to DNA synthesis, it can result in the indirect formation of SSBs (Caldecott, 2003). SSB are detected by poly(ADP-ribose) polymerases (PARPs), which are activated in response to both direct and indirectly formed SSBs (Caldecott, 2014a). Active PARPs and poly(ADP)-ribosylation (PARylation) recruit SSB repair proteins for efficient repair.

Because SSBs and DSBs are repaired through different mechanisms, these damage pathways are often viewed separately. However, DSBs can be thought of as two closely-spaced SSBs on opposite strands that cannot be repaired by SSB repair (Jeggo and Löbrich, 2007), while SSBs can become DSBs through both polymerase run-off or from replication fork collapse resulting from replication stress. Therefore, it is possible that there is some overlap between the initial cellular responses, for example, some DSBs might be directly sensed by PARP enzymes. It is important to note that despite the huge body of work, there is no definitive consensus on a universal DDR signalling mechanism(s) generally accepted in the field. However, emerging evidence supports PARP and PAR-dependent signalling in some DSB responses (see below). However, it is still unclear whether PARP-dependent PARylation, vital for SSB repair, must be removed prior to DSB repair, or if it plays an active role in DSB response (Caldecott, 2014b; Chaudhuri and Nussenzweig, 2017). In addition, the recruitment of other DNA damage sensors, including KU70/80 and MRN, is also highly complex. The non-focal response to DSB that occurs within seconds of DSB formation constitutes the Immediate-Early response (Figure 1B), which also includes ‘fast-kinetic’ canonical non-homologous end joining (cNHEJ), which does not require further signal transduction.

The signal transduction inherent to the Early response to many DSBs is largely carried out by ataxia-telangiectasia mutated (ATM) protein kinase (see below, Figure 1C, and Savitsky et al., 1995; Ziv et al., 1997; Khanna et al., 2001; Shiloh, 2003; Falck et al., 2005; Maréchal and Zou, 2013). At DSBs, ATM is the apical kinase, phosphorylating many substrates and triggering complex downstream post translational modifications (PTMs), including additional phosphorylation events, as well as methylation, ubiquitination (also known as ubiquitylation), neddylation, fatylation, ufmylation, and sumoylation of substrates (Matsuoka et al., 2007; Mu et al., 2007; Bensimon et al., 2010; Dou et al., 2011; Brown and Jackson, 2015; Yu et al., 2020).

The signal cascade of the Immediate-Early and Early responses to DSBs leads to the recruitment of the scaffolding proteins 53BP1 and BRCA1 in the Late response, which is characterised by the precisely regulated balance between end resection and end protection promoted by these complexes (Figure 1D). After 53BP1 and BRCA1 are recruited, a DSB is committed to a specific repair pathway by mechanisms that are under intense study (Figure 1E–I). There are then two major pathways for DSB repair. One, termed ‘slow-kinetic’ canonical non-homologous end joining (slow-kinetic cNHEJ), directly aligns and ligates the broken DNA ends, with minimal or no DNA polymerase activity required (Figure 1E and Chang et al., 2017; Ronato et al., 2020; Zhao et al., 2020). It is active throughout the cell cycle and requires highly limited resection (0–5 nt) by the nuclease Artemis. The other, termed homologous recombination (HR), requires a homologous sequence for templated DNA synthesis to effect DSB repair (Figure 1I and Li and Heyer, 2008; Ronato et al., 2020). For non-repetitive DNA this is typically the sister chromatid, which is only available after DNA synthesis has occurred. For repetitive sequences, homologous sequences are available *in cis* for HR repair throughout the cell cycle. A good example of this is repair of DSBs within ribosomal DNA (van Sluis and McStay, 2017), and more recently with centromeric DNA (Yilmaz et al., 2021).

While DSB repair pathways have been extensively reviewed, particularly with a focus on the repair mechanisms (Panier and Boulton, 2013; Ceccaldi et al., 2016; Krenning et al., 2019; Scully et al., 2019; Mirman and de Lange, 2020; Ronato et al., 2020), here we use the terminology Immediate-Early, Early, and Late responses, borrowed from viral regulatory proteins (Everett, 1987), to facilitate an integrated description of the cell’s complex responses to DSBs prior to repair itself. ‘Immediate-Early’ responses include initial DSB sensing, while the ‘Early’ response is characterised by chromatin changes and ATM signalling, and the ‘Late’ response includes pathway choice prior to repair. A distinguishing characteristic between these divisions is that the Immediate-Early proteins do not form large easily discernible foci, while most Early and Late response proteins do show such focal recruitment. The nature of these foci is the topic of much debate; they have been described as liquid-liquid phase-separated condensates, droplets, biomolecular condensates, or membrane-less organelles (Fijen and Rothenberg, 2021). Precisely how these Immediate-Early, Early, and Late pre-repair responses tie into actual DSB repair remains unclear. Here, we present a discussion of the roles of PARPs, KU70/80, MRN, and fast-kinetic cNHEJ in the ‘Immediate-Early’ response; how chromatin alteration and ATM regulate the ‘Early’ response; the critical role of 53BP1 and BRCA1, as well as ongoing roles for KU70/80 and MRN in the ‘Late’ response; and briefly consider downstream DSB repair.

## The Role of PARP in the Immediate-Early Double Strand Break Response

**FIGURE 2:**
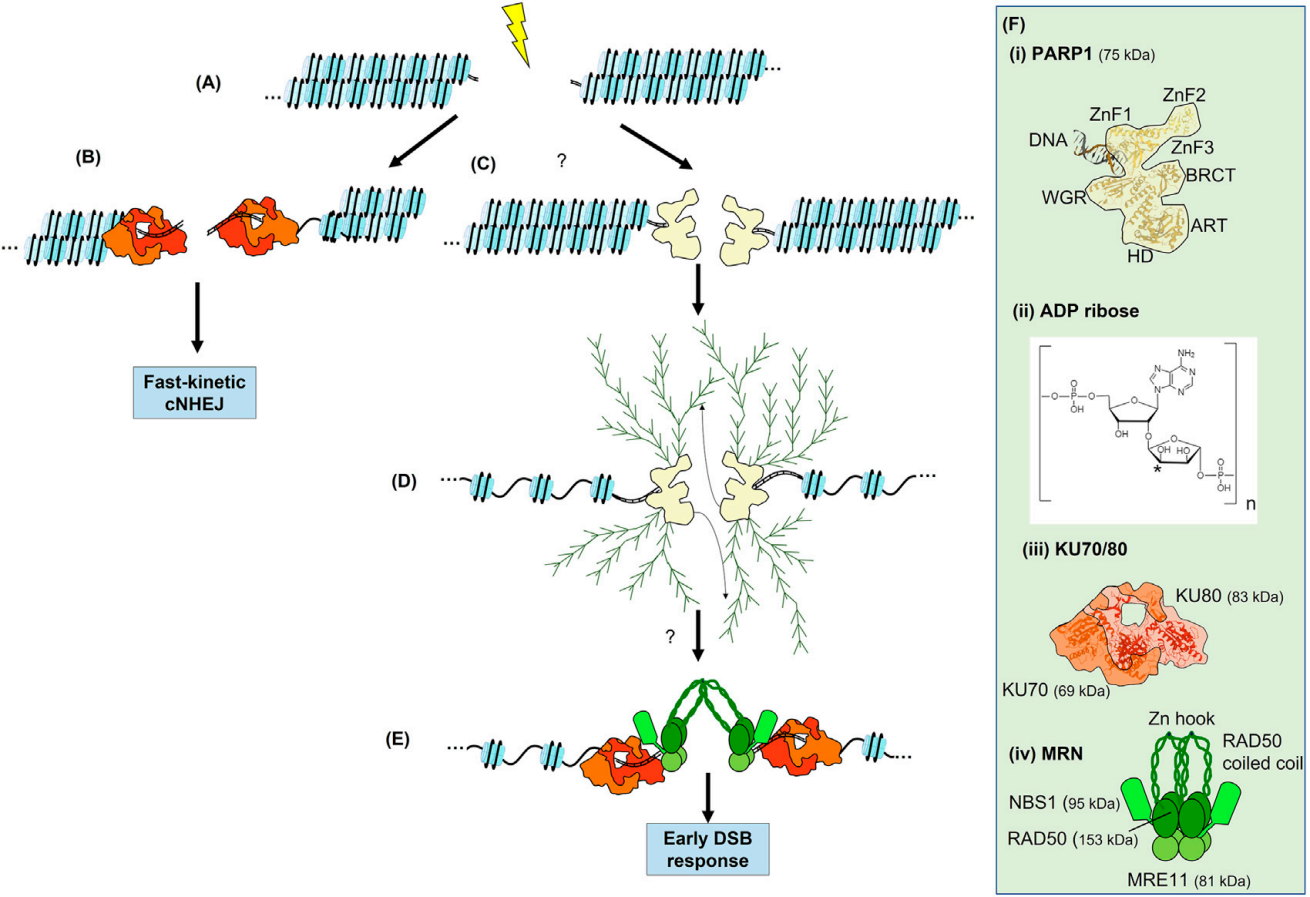
The Immediate-Early response to a DSB. **(A)** Generation of a DSB within a chromatin fibre. **(B)** Recruitment of KU70/80 to some breaks. If KU70/80 is recruited and the break is easily ligatable, fast-kinetic cNHEJ can occur. **(C)** The recruitment of poly(ADP-ribose) polymerases (PARP) to some DSBs. **(D)** Expansion of linear and branched chains of polyADP ribose (PAR) on PARP results in chromatin decondensation. PARylation of other proteins occurs but is not shown. PARylation is a highly dynamic process involving reversal of PARylation by poly(ADP-ribose) glycohydrolase (PARG, not shown). Many DNA repair proteins are recruited to both PARP and PAR chains. The involvement of PARP in the DSB response is not fully elucidated. **(E)** Recruitment of both KU70/80 and MRN are promoted by PARP and PARylation at some DSBs. Initial recruitment of these complexes to the vicinity of the DSB is via interaction with PAR, subsequently they directly bind DSB ends and contribute to synapsis. KU70/80 appears to be recruited with faster kinetics than MRN and is required for fast-kinetic cNHEJ during the Immediate-Early response as seen in **(B)**. Note that both KU70/80 and MRN are required for slow-kinetic cNHEJ but KU70/80 must be evicted prior to MRN-dependent HR. **(F)** Structures of PARP1, ADP-ribose, KU70/80 and MRN. Where known, structure is superimposed within overall architecture as illustrated. **(i)** PARP1, the N-terminal zinc fingers, ART, HD and WGR domains of PARP are indicated (see text for details), as well as the interaction with a DSB (Langelier and Pascal, 2013; Beek et al., 2021). The interaction with HPF1 is not shown. **(ii)** ADP-ribose, linear chains are polymerised via the phosphate groups, whereas the asterisk indicates the position of at which branched chains are attached (Drenichev and Mikhailov, 2016). **(iii)** KU70/80 heterodimer shown in shades of red (Walker et al., 2001). **(iv)** MRN complex in shades of green with the coiled-coils of RAD50 extending outwards (Williams et al., 2009; Casari et al., 2019). In **(D)** these coiled-coils interact via their zinc hooks.

The role of PARPs and PARylation in the DSB response is still under debate (Yang et al., 2004; Wang et al., 2006; Patel et al., 2011; Langelier et al., 2012; Caldecott, 2014b; Fouquerel and Sobol, 2014; Strickfaden et al., 2016; Pascal, 2018; Yang et al., 2018; Caron et al., 2019; Murata et al., 2019). While the early literature focussed upon the role of PARPs in SSB repair, it has become more widely implicated in other branches of the DDR. This is largely due to the structure of some DSBs, in which two SSBs on opposites strands of DNA occur near enough that the two ends can separate (Figure 2A). There is emerging data showing that PARP1, PARP2, and PARP3 also function at DSBs. This includes structural data showing that PARP1 binds to DSBs (Langelier et al., 2012), as well evidence that PARP1 and KU70/80 compete for DSBs (Figure 2B, Wang et al., 2006; Yang et al., 2018), that PARP1 negatively regulates resection (Caron et al., 2019), that defective cNHEJ contributes to the sensitivity to PARP inhibitors (Patel et al., 2011), that PARP3 accelerates cNHEJ (Rulten et al., 2011), and, finally, evidence that PARP1 and KU70/80 can form a complex together (Galande and Kohwi-Shigematsu, 1999). However, it is likely that the linkages between PARPs and DSB responses can be confounded by fast-kinetic cNHEJ functioning in competition with PARP responses during the Immediate-Early response, whereas slow-kinetic cNHEJ that occurs after the Late response appears to be promoted by PARP and PARylation (see discussion on fast- and slow-kinetic cNHEJ in the next section). Important additional considerations are evidence that PAR-dependent regulation of chromatin remodelling enzymes is required to propagate the DSB signals (Strickfaden et al., 2016); that PARP and PARylation can directly recruit specific DSB repair proteins (discussed below); and, lastly, that the consumption of NAD^+^ by PARPs and production of ATP by PARG leads to metabolic shifts that promote specific repair outcomes (Fouquerel and Sobol, 2014; Murata et al., 2019).

PARPs have been extensively studied in the context of SSB repair and BER (Chambon et al., 1963; Benjamin and Gill, 1980; Caldecott, 2014b). There are seventeen members of the PARP family in humans; most of these primarily add a mono(ADP-ribose) to their target proteins (Beek et al., 2021). The mono(ADP-ribose), termed MAR, is most often added to the side (R) carboxyl groups of glutamate and aspartate via an ester bond, but can also be added to the R groups of cysteine and lysine (Wei and Yu, 2016). PARP1 and PARP2 have largely overlapping functions in the DNA damage response, although PARP1 is most prevalent, accounting for 80%–90% of the PARylation in response to strand breaks (Caron et al., 2019). PARP3, which adds mono(ADP-ribose) groups, has also recently been identified as a regulator in the DSB response (Beck et al., 2014). Thus, although PARP1 is the major player, PARP1, PARP2, and PARP3 are collectively responsible for the emerging roles of PARP in the DSB response through their auto-PARylation and PARylation of downstream targets (Figures 2C,D and Wei and Yu, 2016). Although the structure of the individual domains of PARP1 have been solved, its complete structure remains elusive (Figure 2F). Three zinc-finger (ZnF) domains compose the N-terminal, the first two of which are homologous and recognize DNA (Langelier and Pascal, 2013). Next, the BRCA1 C-terminal (BRCT) domain mediates PAR-dependent protein-protein interactions. The WGR domain is named after its conserved amino acid sequence, and is also involved in DNA binding (Beek et al., 2021). Finally, the catalytic domain, which binds NAD^+^ and catalyses addition of ADP-ribose, is comprised of a helical subdomain (HD) and an ADP-ribosyl transferases (ART) subdomain. Interestingly, PARP2 and PARP3 lack the N-terminal ZnF1-3 and BRCT domain present in PARP1 but are still able to bind to DNA via the retained WGR domain.

PARP1/2 are nuclear and depend upon an accessory factor, histone PARylation factor 1 (HFP1), for their activity (Krüger et al., 2020; Suskiewicz et al., 2020). Due to the conformation of the domains of PARP1/2 around the broken DNA end, PARP1/2 preferentially adds PAR to itself via auto-modifications. However, when HPF1 is bound near the PARP1/2 active site, it provides a new catalytic amino acid, N285, that allows PARP1/2 to target serine residues rather than aspartate and glutamate residue targets, contributing to PARP1/2’s activity of initiating and elongating the PAR chains. Interestingly, although HPF1 is expressed at relatively low, it appears to be only needed at a ratio of 1:50 to switch the activity of the highly abundant nuclear protein PARP1 (Langelier et al., 2021).

PARP1 is the earliest known protein that senses DNA strand breaks, and binds rapidly to free DNA ends through its DNA-binding domain (DBD) (Ali et al., 2012). It accumulates at lesions in as little as half a second post photoinduced irradiation, and peaks around 5 s (Haince et al., 2008). While the rapid localisation of PARP1 to single-strand breaks has been well characterised, its precise mechanism of localisation to DSBs remains unknown (Liu et al., 2017)*.* In SSB repair, PARP1 and its associated PARylation events recruit X-ray repair cross-complementing protein 1 (XRCC1) to SSB sites, with XRCC1 functioning as a scaffold for the subsequent binding of SSB repair proteins (Masson et al., 1998; Breslin et al., 2015; Hanzlikova et al., 2017; Adamowicz et al., 2021). It is currently unclear how the cell might differentiate between isolated SSBs and those that occur in very close proximity but on opposite strand (i.e., DSBs) after the initial PARylation. One hypothesis is via a possible ‘PAR code’, an emerging model in which the length and branched nature of the PAR chain controls specific protein recruitment, and thus repair pathway choice (Aberle et al., 2020). A second hypothesis is that due to the unique structure of PARP1, which allows it to be allosterically regulated, the type of DNA break itself could determine the type of PAR chain, which in turn could regulate specific DDR protein recruitment (Pascal, 2018). However, it is currently unknown if the structure of PAR chains differs between the SSB and DSB responses (Leung, 2020).

An observation favouring the involvement of PARPs in the DSB response is that many DSB response proteins bind PAR through their BRCT and forkhead-associated (FHA) domains (Leung and Glover, 2011; Li et al., 2013). PAR-dependent recruitment of DSB repair proteins supports a model in which PARPs and PARylation are required for DSB repair, rather than being merely a remnant of failed attempts to repair DSBs using the SSB repair machinery. Importantly, the KU70/80 and MRN complexes can bind to PAR and have been reported to be dependent upon PARP1/2 activity for their recruitment to DSBs (Figure 2E and Haince et al., 2008; Beck et al., 2014; Caron et al., 2019). In addition, PAR interacts with ATM (Aguilar-Quesada et al., 2007), DNA-PKcs (Spagnolo et al., 2012), and BRCA2 (Bryant et al., 2005). PARPs further promote the recruitment of CHD2 (a chromatin remodeller) and BRCA1 (Pascal, 2018). Furthermore, many proteins within the DSB response are targets for PARylation, including RPA (Maltseva et al., 2018), BRCA1 (Li and Yu, 2013), and BARD1 (Li and Yu, 2013). Interestingly, KU70/80 is also PARylated by PARP3 (Beck et al., 2014). The persistence of PARPs and PARylation throughout the Immediate-Early, Early, and Late DSB responses is consistent with a model in which the activity of PARP enzymes is required throughout the DSB response. However, the complex dynamics of PARP1, PARP2, and PARP3 binding to DSBs and the resulting PARylation remain to be fully elucidated and functionally defined.

The rapid PARylation that occurs in the vicinity of a strand break leads to local decondensation of the chromatin, believed to provide space for subsequent protein recruitment (Strickfaden et al., 2016; Pascal, 2018). Of such recruitments, three involve the chromatin remodelling enzymes: amplification in liver cancer 1 (ALC1), and chromodomain helicase DNA binding proteins CHD2 and CHD7 (Luijsterburg et al., 2016; Rother et al., 2020; Verma et al., 2021). CHD7 acetylates histone H4, leading to further chromatin decondensation, facilitating recruitment of histone deacetylase 1 and 2 (HDAC1/2). The ensuing deacetylation of histones leads to recondensation of the chromatin. Together, this expansion and contraction of chromatin comprises a dynamic process sometimes termed ‘chromatin breathing’ (Lans et al., 2012). Chromatin breathing offers a more dynamic and nuanced view of the role of chromatin state in the DSB response, rather than a simpler model in which condensed or open chromatin favours either cNHEJ or HR, respectively. In addition, PARP-dependent expansion of the damaged chromatin has recently been shown to recruit the zinc-finger protein ZNF384, which binds DNA ends *in vitro* and is recruited to DSBs *in vivo* via its C2H2 motif*.* ZNF384 then functions as an adaptor of KU70/80, which promotes the assembly of KU70/80 at DSBs for repair by cNHEJ (Singh et al., 2021).

The metabolic state of the cell is also important to the Immediate-Early DSB response, as PARylation and repair consumes energy. PARylation is a NAD^+^-dependent reaction and depends heavily on cellular metabolism. The reverse reaction, dePARylation, by PARG, recycles some of that ATP. PARG has not yet been as extensively studied as PARP1 in the DDR, but some recent studies indicate that PARG binds to nudix hydrolase 5 (NUDT5) and, as PARG hydrolyses the PAR chains, NUDT5 is able to convert the ADP-ribose into ATP, providing energy for downstream processes (Wright et al., 2016). Perhaps the roles of PARG in the Immediate-Early DSB response could be as diverse as those of PARP itself (Feng and Koh, 2013), and future work will be needed to fully decipher its role in DSB repair (Feng and Koh, 2013).

While PARP and KU70/80 have been reported to form a complex (Galande and Kohwi-Shigematsu, 1999), they have also been reported to be mutually exclusive at some lesions (Wang et al., 2006). The latter study supports competition between PARP and KU70/80 at DSBs, but also describes an ‘alternate’ NHEJ pathway, that is sensitive to PARP inhibitors, involving the core cNHEJ factors as well as Artemis. We interpret this ‘alternate’ pathway to be what has now been termed slow-kinetic cNHEJ. Another report is consistent with both PARP and KU70/80 being recruited to breaks earlier than other DSBs sensors (Yang et al., 2018). Surprisingly, they report that only KU70/80 binds to DSBs in G1, while in S/G2 both KU70/80 and PARP compete for binding with PARP regulating KU70/80 removal. On the other hand, PAR-dependent recruitment of KU70/80 to DSBs and, this time, retention has been reported in *Dictyostelium discoideum* (Couto et al., 2011). Importantly, this study provides evidence for evolutionarily conservation of PARP function in some cNHEJ repair. Yet another study shows that PARP1 has a role in resection, and that loss of PARP1 results in hyper-resection as well as loss of KU70/80, 53BP1, and RIF1 consistent with PARP having functions upstream of slow-kinetic cNHEJ and HR (Caron et al., 2019). Collectively, these data implicate PARPs in DSB repair, although inconsistencies remain to be resolved. Perhaps some of the contradictory results can be rationalised by the division of cNHEJ into its fast- and slow-kinetics subpathways, PARP-independent and -dependent, respectively (see below).

In summary, SSBs and at least some DSBs appear to require PARPs and the associated PARylation for their repair. Contradictory data on the role of PARPs in DSB responses is a source of confusion in the field, but despite this, PARP and PARylation likely constitutes the initiation of the Immediate-Early response to some DSBs. The ensuing chromatin relaxation and PAR-dependent recruitment of chromatin remodellers and other factors can lead the recruitment and activation of further downstream DSB response proteins.

## The Role of KU70/80 and MRN in the Immediate-Early Double Strand Break Response

KU70/80 and MRN recruitment are also part of the Immediate-Early response (Figures 2B,E). MRN and KU70/80 are frequently considered as DSB sensing proteins. However, if we consider a ‘sensor’ to be the initial detection of DSBs, this can be misleading as both MRN and sometimes KU70/80 are loaded subsequent to initial PARylation, and their recruitment can be dependent upon PARP activity (Caron et al., 2019; Ingram et al., 2019). However, if a ‘sensor’ is more broadly defined as a protein that binds directly to DNA lesions (Jackson, 2002), then PARP, MRN, and KU70/80 can all be counted as DSB sensors. In addition to the complex competition and recruitment interactions between KU70/80 and PARP, KU70/80 and MRN also share what has been termed ‘entwined’ loading, meaning they are not loaded in defined sequential order or competitively, but rather with more complex dynamics that include multiple points of crosstalk (see below and Rupnik et al., 2008; Shibata et al., 2018; Ingram et al., 2019). In addition, the common model where KU70/80 solely promotes NHEJ by recruiting DNA-PK, and MRN promotes HR by recruiting ATM, is clearly an oversimplification, as both complexes can be loaded to the same DSB (Britton et al., 2013; Ingram et al., 2019; Qi et al., 2021).

The KU70/80 heterodimer is composed of two subunits, 69 and 83 kDa, respectively, forming an open ring around DNA ends (Figure 2F and Walker et al., 2001; Jackson, 2002). The major portion of the KU70/80 complex cradles the DSB, effectively covering one surface of the DNA helix but leaving the other surface more open to allow recruitment of further end joining proteins. Once bound, KU70/80 not only facilitates synapsis but also protects DNA ends from resection, thereby promoting cNHEJ. Emerging data supports a division of cNHEJ into two distinct biphasic pathways, termed fast-kinetic and slow-kinetic cNHEJ (Figures 1B, 2B,E, and Jakob et al., 2011; Biehs et al., 2017; Chang et al., 2017; Löbrich and Jeggo, 2017; Shibata et al., 2018; Frit et al., 2019; Setiaputra and Durocher, 2019; Shibata and Jeggo, 2020a; Shibata and Jeggo, 2020b; Qi et al., 2021). The fast-kinetic cNHEJ is also termed 53BP1-, Artemis-, or resection-independent cNHEJ, with Artemis clearly function downstream of ATM (Riballo et al., 2004; Woodbine et al., 2011). It relies upon the essential core cNHEJ factors KU70/80, DNA-PKcs, XRCC4, XLF, and LIG4, which do not form detectable foci during the Immediate-Early response. Fast-kinetic cNHEJ repair likely repairs low complexity breaks that are easily ligatable, and is estimated to repair around 70%–80% of DSBs resulting from X-ray irradiation throughout the cell cycle. KU70/80 appears to be recruited within a second of PARP1, while initial recruitment of MRN is typically in the range of tens of seconds later (Mari et al., 2006; Haince et al., 2008; Liu et al., 2017; Yang et al., 2018). Their rapidity of recruitment and high nuclear abundance likely makes the kinetics of protein recruitment difficult to study during the Immediate-Early response, as visible foci do not form. The Immediate-Early loading of KU70/80 directs easily repairable DSB towards highly efficient fast-kinetic cNHEJ, likely coinciding with recruitment of PARPs to some breaks, and prior to subsequent loading of MRN. However, it remains unclear how fast-kinetic cNHEJ ties into the nature of KU70/80 and MRN loading, specifically when both complexes are loaded onto the same DSB (Britton et al., 2013; Ingram et al., 2019).

MRN consists of a hetero-hexameric complex consisting of two molecules each of MRE11, RAD50, and NBS1, although there is some discrepancy over whether one or two monomers of NBS1 are associated (Figure 2F and Paull, 2018; Syed and Tainer, 2018; Tisi et al., 2020). The MRN complex changes conformation upon ATP binding, enabling MRE11 to span both sides of the DSB, with the coiled-coil domain of RAD50 bridging the gap between the DNA ends. MRE11 is a short-range exonuclease that chews back DNA in a 3′ to 5′ direction, revealing short ssDNA tracts. It also has endonuclease activity important for bypassing blocked DSB ends. Except for fast-kinetic cNHEJ, which does not require resection or processing, tracts of ssDNA are required for all remaining DSB repair pathways; hence MRN is not likely to be critical for fast-kinetic cNHEJ. Consistent with a later function for MRN, the most important role of NBS1 appears to be subsequent binding to ATM during the Early response (Wu et al., 2012; Tisi et al., 2020). Additionally, recruitment of multiple MRN molecules to DSBs has been shown *in vitro*, and proposed to contribute to synapsis in a process that has been termed ‘molecular velcro’ (De Jager et al., 2001; Rupnik et al., 2009). MRN may initially be recruited to the immediate vicinity of DSBs via an interaction with PAR chains, although its initial recruitment could also be due to its ‘facilitated diffusion’ capabilities, in which MRN can localise to DNA via RAD50-dependent scanning of DNA for broken DNA ends, which are then recognised by MRE11 (Myler et al., 2017).

The recruitment of KU70/80 within seconds of PARP1 suggests a causal relationship, and an interaction between PARP1 and KU70/80 has been reported, although the detailed mechanism of by which KU70/80 and PARP1 crosstalk with each other remains unclear (Galande and Kohwi-Shigematsu, 1999; Isabelle et al., 2010; Liu et al., 2017; Caron et al., 2019)*.* A possible point of insight is that KU70/80 has been reported to be a PARylation target of PARP3, albeit PARP3 plays a more minor role than PARP1 in the DDR (Beck et al., 2014). New data also supports that PARP-dependent chromatin decondensation facilitates KU70/80 loading via ZNF384 binding (Singh et al., 2021). The Immediate-Early recruitment of MRN complex, which is slightly later than KU70/80, is likely to be at least partially explained by the ability of NBS1 to recognise PARylation (Haince et al., 2008). Whether MRN and KU70/80 load onto the same break, the relative order of this loading and whether they both persist throughout the DSB response is the subject of debate (Hartlerode et al., 2015; Ingram et al., 2019; Paull, 2021), although *in silico* modelling has supported a so-called ‘entwined pathway’ in which there are multiple point of crosstalk between KU70/80 and MRN loading as opposed to competitive or sequential loading (Ingram et al., 2019).

In summary, if the break is easily ligatable, KU70/80-dependent repair fast-kinetic cNHEJ occurs (Figure 2B). KU70/80 and MRN complexes can be recruited during the Immediate-Early response, which is initiated mainly by PARP1 and the subsequent PARylation events and chromatin decondensation (Figures 2C–E). These proteins are damage ‘sensing’ proteins in the sense that they bind directly to the DNA damage. It is possible that there are other proteins that fit this definition, such as the recently reported SIRT6 (Onn et al., 2020). Although MRN and KU70/80 can both be loaded together at a single DSB, only KU70/80 is needed for fast-kinetic cNHEJ. However, MRN’s end processing activities and the recruitment of ATM are required for both slow-kinetic cNHEJ and HR, and KU70/80 is likely retained at breaks reapired by slow-kinetic cNHEJ.

## The Early Response to Double Strand Breaks Is Chromatin-Based

**FIGURE 3:**
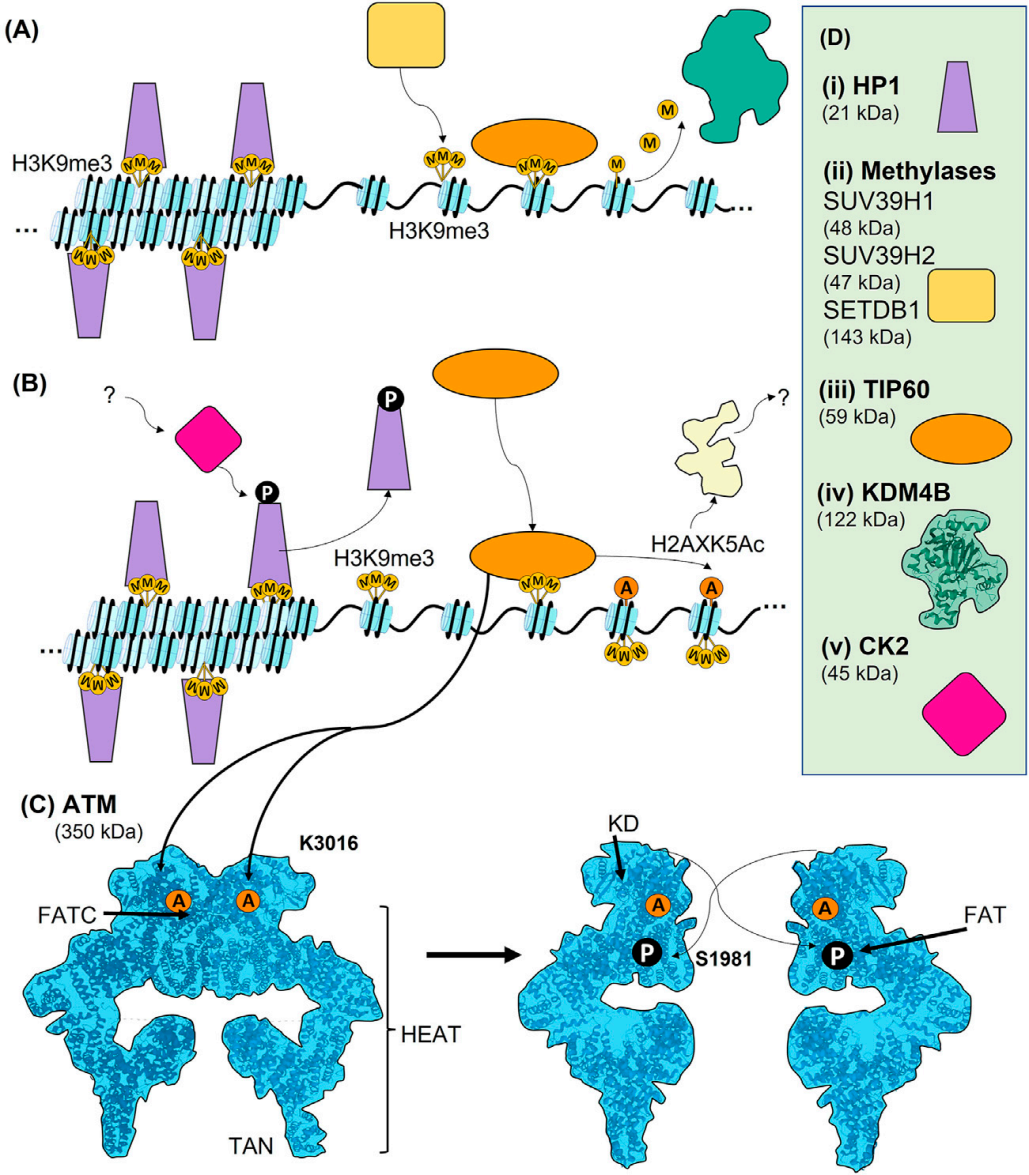
Early chromatin events leading to the activation of ATM. **(A)** The compaction of chromatin prior to DNA damage involves trimethylation of histone H9 at lysine 9 (H3K9me3). In heterochromatin, HP1 binds to H3K9me3 and contributes to chromatin condensation. Although a largely heterochromatic mark, H3K9me3 is also present in euchromatin, where it contributes to transcriptional regulation, and is therefore tightly regulated. It can be methylated by SUV39H1, SUV39H2, and SETDB1, and demethylated by KDM4B. When free of other binding proteins H3K9me3 can bound by TIP60 in its role as a regulator of transcription. **(B)** The regulation of H3K9me3 upon DNA damage and the activation of TIP60. In heterochromatin, CK2-dependent phosphorylation of HP1 (T51) causes its release from H3K9me3, leading to chromatin decondensation. Free H3K9me3 can then be bound by TIP60. When TIP60 is bound it can acetylate lysine 5 of histone H2AX (H2AXK5Ac), leading to further chromatin decondensation. H2AXK5Ac can also contribute to PARP-dependent PARylation around DSBs. **(C)** Activation of ATM occurs via TIP60-dependent acetylation of K3016 within the FATC domain of ATM. ATM is present as a largely inactive dimer prior to damage, and this acetylation causes it to monomerise. Autophosphorylation of S1981 within the FAT domain of ATM also likely contributes to activation of ATM. In addition to the FATC and FAT domain, ATM also is comprised of a kinase domain (KD) and HEAT repeats. Both the FAT and TAN domain are specialised HEAT repeats. **(D)** Where known, structure is superimposed within overall architecture as illustrated. (i) Schematic of HP1. (ii) General schematic of H3K9 methylases, including SUV39H1, SUV39H2, and SETDB1. (iii) TIP60 schematic. (iv) CK2 schematic.

In addition to events at the DSB, parallel chromatin-based responses occur both proximally and distally to the DSBs. Separately from the Immediate-Early DSB sensing events discussed earlier, the chromatin-based Early response to DSBs revolves around the trimethylation of histone H3 K9 (H3K9me3) (Figure 3). Regulation of this histone modification by proteins such as heterochromatin protein 1 (HP1, also termed chromobox protein homolog 1) and Tat-interactive protein 60 kDa [TIP60, also termed lysine (K) acetyl transferase, KAT5] centres on ATM activation. TIP60 is the acetyltransferase component of the multicomponent NuA4 complex, which acetylates lysines in multiple targets, including ATM and histone H4, and is important for transcription and DNA repair, as well as contributing to histone exchange (Lee and Workman, 2007; Price and D’Andrea, 2013; Jacquet et al., 2016).

Interestingly, the state of chromatin condensation plays an important role in activating and maintaining the DSB responses leading to DSB repair. Condensed chromatin is regulated by the binding of HP1 (Ayoub et al., 2008; Becker et al., 2016; Kumar and Kono, 2020). HP1β is the most abundant isoform of HP1, while the HP1α and γ variants play lesser roles in chromatin condensation (Kumar and Kono, 2020). HP1β binds to the H3K9me3 heterochromatin marker to maintain the condensed chromatin state. However, the H3K9me3 mark is also present in euchromatin prior to damage, where the level of H3K9 methylation is maintained by a combination of methylases and demethylases (Figure 3A). Methyltransferases include suppressor of variegation 3–9 homolog 1 and 2 (SUV39H1 and SUV39H2), and SET domain bifurcated histone lysine methyltransferase 1 (SETDB1) (Figure 3D and Monaghan et al., 2019). The demethylases include a family of proteins called lysine (K) demethylases 4 (KDM4A, also termed JMJD2A) that act as demethylases of H3K9me2/3 (Mallette et al., 2012). Interestingly, KDM4B recruitment is promoted by PARP (Khurana et al., 2014), suggesting crosstalk with the Immediate-Early response. Importantly, the level of histone H3K9 methylation is in constant flux dependent upon the cell cycle and the specifics of hetero- or euchromatic packaging (Sulkowski et al., 2020).

In heterochromatin after damage, HP1β is phosphorylated (on T51) by casein kinase 2 (CK2), causing its displacement from H3K9me3 (Figure 3B and Ayoub et al., 2008). CK2, which is functionally highly pleiotropic in cellular signalling, also phosphorylates multiple other targets in the DDR, although its precise regulation and functions in the DDR are unclear. For example, it is not known how CK2 is activated upon DNA damage to phosphorylate HP1β on residue T51 (Ayoub et al., 2008). Although primarily a mark of heterochromatin, H3K9me3 also functions within euchromatin as a regulator of transcription. DSBs within euchromatin result in a rapid spike of H3K9me3 in the chromatin flanking the DSB, although precisely how this is regulated is not well understood. It is possible that because KDM4B constantly removes H3K9 methylation, its inhibition at DSBs would allow for a quick and local increase in H3K9 methylation (Sulkowski et al., 2020). Another possibility causing this rapid increase in H3K9me3 upon DNA damage is a pathway that involves ufmylation, a ubiquitin-like protein, of histone H4 at K31 (H4K31Ufm) by ULF1, which is recruited by the MRN complex. H4K31Ufm is read by serine/threonine-protein kinase 38 (STK38) and somehow facilitates recruitment of SUV39H1 to breaks, which then locally trimethylates H3K9 (Qin et al., 2019; Qin et al., 2020). However, this ufmylation-dependent pathway is thought to be more likely an MRN-dependent positive feedback loop and does not account for the initial spike of H3K9me3, but rather its spreading and subsequent activation of ATM (Qin et al., 2019; Qin et al., 2020). Upon HP1β displacement, the histone acetyltransferase TIP60 can bind, via its chromodomain, to H3K9me3 (Sun et al., 2009). It is interesting that although HP1β must be removed, it has also been shown to be recruited to DSBs via an unclear mechanism that involves its chromoshadow domain, suggesting an additional function in the DSB response (Luijsterburg et al., 2009). TIP60 can also bind to H3K36me3, and together, these two chromatin marks act as allosteric regulators of TIP60 acetyltransferase activity (Figure 3B and Bakkenist and Kastan, 2015).

Once the H3K9me3 histone mark is revealed within heterochromatin or generated within euchromatin, TIP60 binds H3K9me3 and acetylates K3016 of ATM (Figure 3C and Sun et al., 2010; Bakkenist and Kastan, 2015). In fact, ATM and TIP60 can form a stable complex through the FATC domain of ATM, and this interaction is likely what brings TIP60 into proximity with K3016, allowing the acetylation. The TIP60-ATM interaction appears constitutive, although TIP60’s histone acetylation activity and the kinase activity of ATM are indeed damage-dependent (Sun et al., 2005). Acetylation of ATM, which is present as an inactive dimer in the nucleus prior to damage, causes ATM to monomerise and autophosphorylate on residue S1981. It is not currently known if this phosphorylation event is *in cis* or *in trans*, or if this phosphorylation is necessary for activation of ATM or just a marker of active ATM (Bakkenist and Kastan, 2003; Zong et al., 2015; Burger et al., 2019). In fact, ATM has been reported to have several other sites of autophosphorylation, which likely play roles in DSB repair yet to be elucidated (Kozlov et al., 2006; Kozlov et al., 2011). At DSBs, ATM phosphorylates many substrates, resulting in complex signal transduction involving numerous distinct PTMs (Matsuoka et al., 2007; Mu et al., 2007; Bensimon et al., 2010; Dou et al., 2011; Brown and Jackson, 2015; Yu et al., 2020). Thus, the TIP60 acetylation-dependent monomerisation of ATM and its likely phosphorylation-dependent activation leads to an extensive leads to an extensive signal transduction network in the Early DSB response (see below).

It is intriguing to note that PARP1/PARylation could aid in recruiting TIP60 and ATM to sites of damage, as ATM binds PARP1 in a PAR-dependent manner (Aguilar-Quesada et al., 2007; Chaudhuri and Nussenzweig, 2017). ATM has also been shown to be activated by treatments that do not directly cause DNA damage but induced global decondensation of chromatin in the absence of any detectable DNA damage (Bakkenist and Kastan, 2003). Thus, in addition to MRN-dependent ATM recruitment, PAR-dependent chromatin decondensation that occurs at DSBs could also contribute to ATM activation and subsequent recruitment. The activation of ATM creates a positive feedback loop that is dependent upon chromatin decondensation and driven by the binding of TIP60 to H3K9me3, which is either revealed by release of HP1β or promoted by damage-induced formation of H3K9me3 proximal to the DSB. TIP60 also acetylates H2AX on lysine 5 (H2AXK5Ac) in the chromatin proximal to a DSB, promoting PARP1-dependent PARylation, which in turn could contribute to the dynamic chromatin decompaction believed to facilitate DNA metabolic activities at DSBs (Figure 3B and Ikura et al., 2016). In fact, this study showed that PARP1 was part of the TIP60 complex, which is a potential link between the Immediate-Early PARP response and the chromatin-based Early response to DSBs. PARP1, and no doubt PARG, may have functions throughout the DSB response, although, apart from its Immediate-Early functions, the multiple potential roles of PARP1 during the Early and Late responses remain to be defined.

There are other forms of chromatin reorganisation that take place in response to DSBs, such as removal or sliding of nucleosomes, as well as histone exchange (Price and D’Andrea, 2013; Pessina and Lowndes, 2014; Dhar et al., 2017). Pathway choice depends on chromatin state, as for resection to occur, the DNA must be accessible to the resection machinery. Note, however, that similarly to other DNA metabolic transactions such as transcription and replication, nucleosomes may not have to be physically removed for resection to occur. There are many histone modifications that promote or impede resection (Clouaire and Legube, 2019). In general, the balance between such histone modifications affects the binding of factors required for either limited or more extensive resection, which in turn impacts upon pathway choice and fidelity of repair. Specifically within active transcription units, H3K36me3-dependent recruitment of Lens epithelium-derived growth factor p75 splice variant (LEDGF) promotes HR by damage-induced recruitment of CtIP and subsequently the other proteins required for extensive resection (Daugaard et al., 2012; Aymard et al., 2014).

Additionally, ATM-dependent phosphorylation of the RNF20-RNF40 heterodimer, an E3 ubiquitin ligase, results in monoubiquitination of H2B (H2BK120ub1) and the consequent decondensation of the chromatin around DSBs (Moyal et al., 2011). In undamaged cells this monoubiquitination of H2B is normally associated with transcription elongation, but upon damage contributes to the further relaxation of the chromatin flanking DSBs to facilitate recruitment of both NHEJ and HR proteins. Similarly, the ATM-dependent phosphorylation of KAP-1 on S824 leads to decondensation of heterochromatin (Goodarzi et al., 2011). Interestingly, DSBs within heterochromatin relocate to the periphery of the heterochromatic clusters where they can be more easily repaired (Jakob et al., 2011; Hausmann et al., 2018; Clouaire and Legube, 2019).

Together, the highly complex regulation of a multitude of histone modifications in the chromatin flanking DSBs contributes to the activation of ATM and its downstream targets, as well as contributing significantly to downstream pathway choice. While initiated during the Early response, the dynamics of chromatin modification are fluid, continuously adjusting to the specific circumstances of each DSB throughout the entire DSB response.

## The Role of ATM in the Early Double Strand Break Response

**FIGURE 4:**
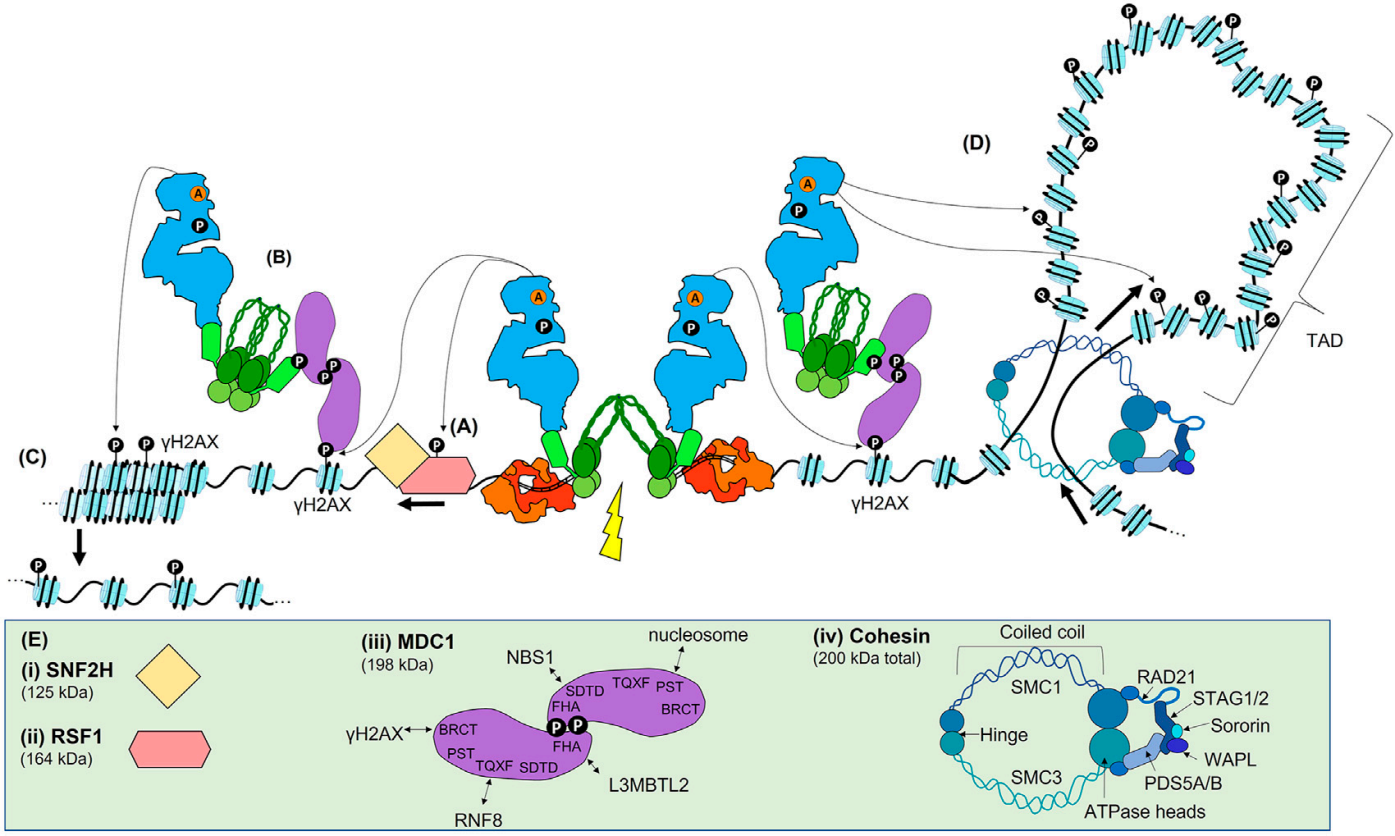
ATM signalling in the Early DSB response. **(A)** Once active, ATM is recruited to sites of DSBs by an interaction with NBS1, where it phosphorylates many target proteins. One of the earliest targets is RSF1, which leads to nucleosome sliding to reveal the DNA surrounding the break. **(B)** ATM phosphorylates H2AX (γH2AX), which allows the scaffold MDC1 to bind via its BRCT domain. As MDC1 is constitutively bound to MRN, the recruitment of MDC1 recruits further MRN and ATM. **(C)** ATM propagates γH2AX via continued MCD1, MRN, and ATM recruitment, leading to chromatin relaxation. **(D)** In addition to this method of γH2AX propagation, γH2AX may also be spread via proposed ‘loop extrusion’ mechanism. In this model, the DSB machinery blocks one direction of the normal loop extrusion that leads to the formation of TADs. As nucleosomes are extruded, ATM phosphorylates H2AX within a given TAD. **(E)** Schematics or structures are shown to the extent of current data. **(i)** and **(ii)** schematic of RSF1 and SNF2H. **(iii)** MDC1 contains many SQ/TQ sites that are phosphorylated by ATM and are required for protein binding. The FHA domain allows for formation of head-to-head dimers of MDC1, and also contributes to L3BMTL2 binding. The SDTD domain interacts with NBS1. The TQXT domain interacts with RNF8. The BRCT domain interacts with γH2AX. **(iv)** Cohesin is made up of the indicated domains.

**FIGURE 5:**
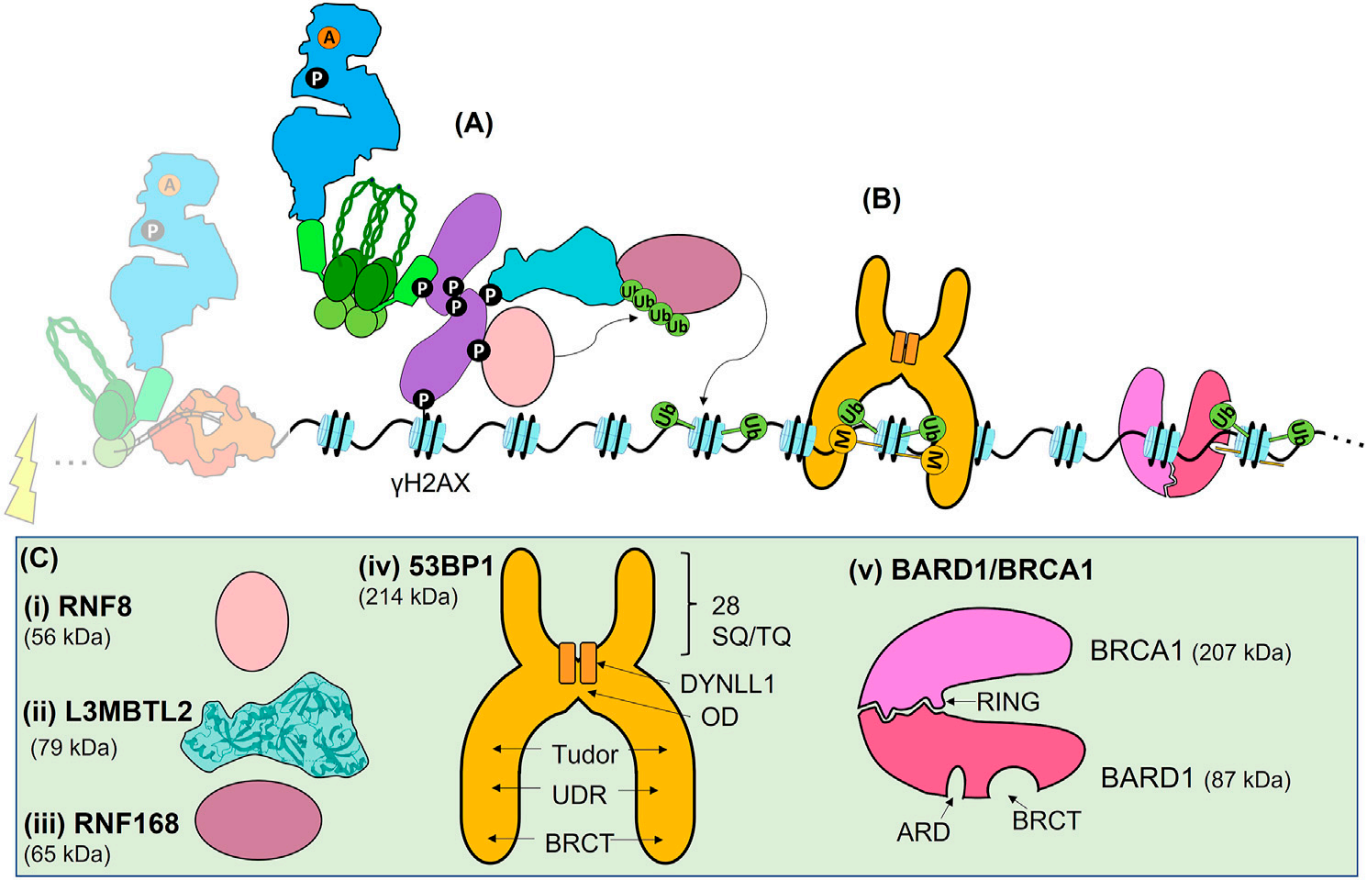
The Early ATM-dependent DSB response results in the ubiquitination of histone H2A(X) (H2A(X)13/15ub) needed for Late protein recruitment. **(A)** DSB signal transduction. The ATM-dependent phosphorylation of MDC1 provides a docking site for the E3 ubiquitin ligase RNF8, and the ATM-dependent phosphorylation of S335 of L3MBTL2 allows it to bind to the FHA domain of MDC1, bringing it in close contact with RNF8. RNF8 polyubiquitinates L3BMTL2 on K659 via K48-linkage, providing a platform for RN168 to bind. RNF168 then monoubiquinates H2A(X)K13/15. **(B)** Together, H2A(X)K15ub and the replication-dependent methylation state of H4K20 recruits either 53BP1 or BARD1, which is in complex with BRCA1. **(C)** Schematics or structures of RNF8, L3BMTL2, RNF168, 53BP1, and BARD1/BRCA1 are shown where known, or informed by known domains where the full structure is not solved. **(i)** RNF8 is an E3 ubiquitin ligase, that interacts with the E2 ubiquitin ligase UBC8 or UBC13 (not shown). **(ii)** L3MBTL2 structure. **(iii)** RNF168 E3 ubiquitin ligase interacts with the UBE2N or UBC13 E2 ubiquitin ligase (not shown). **(iv)** 53BP1 schematic. The UDR domain binds to H4K20me2 and its tandem Tudor domain binds to H2A(X)K15ub. Dimerization of 53BP1 is promoted by the OD and DYNLL1. 28 SQ/TQ sites in the N terminal can be phosphorylated for downstream protein recruitment. **(v)** The ARD domain of BARD1 binds the H4K20me0 mark, while its BRCT domain binds H2A(X)K15ub. BARD1 is in complex with BRCA1. Note that for clarity the DSB and Early ATM signalling proteins are faded out.

Once ATM is activated by TIP60-dependent acetylation, the cell continues with the Early DSB response. The signal transduction pathway initiated by active ATM results in the ubiquitination of histone H2A variants on K13/15 [termed H2A(X)K13/15ub]. The known order of recruitment to chromatin in the vicinity of DSBs is ATM, MDC1, MRN, RNF8, L3MBTL2, and RNF168 (Figures 4, 5 and Salguero et al., 2019). These Early DSB response proteins are notable for their easily visible focal recruitment into micron scale condensates that form around DSBs. A large contribution to the versatility, efficiency, and integrated ‘decision’ making of the DSB repair response is no doubt due to the locally high concentration, ensured by their liquid-liquid phase separation properties, of the many proteins found within foci (Fijen and Rothenberg, 2021).

Once activated (Figure 3), the ATM monomer initiates a phosphorylation cascade involving many transducers and effector proteins including those with roles yet to be defined or those yet to be identified (some of the key proteins are illustrated in Figures 4, 5). Perhaps the first ATM-dependent chromatin event is the phosphorylation-dependent recruitment of the remodelling and spacing factor 1 (RSF1) which is required for reorganisation of the nucleosome(s) immediately proximal to the DSB that is essential for both slow-kinetic cNHEJ and HR (Figure 4A and Helfricht et al., 2013; Min et al., 2014; Pessina and Lowndes, 2014). Another important phosphorylation target of ATM is the MRN complex (Lavin et al., 2015; Syed and Tainer, 2018). MRN bound to broken DNA (Figure 2E) recruits active ATM monomers via an interaction with NBS1, which possibly involves prior K63-linked ubiquitination of NBS1 (Figure 4A and Wu et al., 2012). A proportion of MRN proximal to DSBs during the Immediate-Early response via MRE11-dependent DSB-specific binding DNA, as well as a proportion likely recruited via interaction with PAR (see earlier). However, the dramatic focal accumulation of ATM and MRN in the vicinity of DSBs is chromatin-mediated, rather than directly DNA- or PAR-mediated. Once recruited, ATM phosphorylates H2AX, a variant of histone H2A often found in euchromatin, at residue S139. This PTM, widely known as γH2AX, provides a docking site for MDC1 via its BRCT domain, with MDC1 then acting as a scaffold protein for further protein recruitment, including further ATM and MRN, throughout the remainder of the DSB response (Figure 3B and Jungmichel et al., 2012). In addition to this widely appreciated mechanism of ATM accumulation at DSBs, an additional regulator has been suggested: Pellino1, yet another E3 ubiquitin ligase, that is recruited to the DSB, phosphorylated by ATM, and then binds to γH2AX to further promote the accumulation of ATM and MRN, and subsequently, of MDC1 (Ha et al., 2019).

Prior to damage, a proportion of the MDC1 scaffold is already bound to NBS1 via the SDTD domain of NBS1, requiring CK2-dependent phosphorylation of the SDTD motif in MDC1 (Goldberg et al., 2003; Chapman and Jackson, 2008; Spycher et al., 2008). Once more, the role of CK2 in the DDR is enigmatic, as whether CK2 is regulated to phosphorylate the MDC1’s SDTD domain is unclear. Phosphorylation of the N-terminus of MDC1 regulates its dimerization, which in turn appears to be required for an effective DSB response (Figure 4E and Luo et al., 2011; Liu et al., 2012). Regardless, the initially recruited MDC1 then recruits more MDC1-bound MRN complexes (Figure 4B and Melander et al., 2008; Spycher et al., 2008; Salguero et al., 2019), while additional active ATM monomers are recruited through their interaction with NBS1. The accumulating ATM then propagates γH2AX, spreading across megabases of chromatin domains on either side of the DSB (Figure 4C). Normally, H2AX is phosphorylated by ATM activity, but can also be phosphorylated by DNA-PKcs and ATR (Stiff et al., 2004; Wang et al., 2005; Caron et al., 2015), and increasing levels of γH2AX results in further chromatin decondensation and amplification of the DSB repair signal.

Recent data suggests another potential mechanism, loop extrusion, which could facilitate γH2AX propagation (Figure 4D and Arnould et al., 2021). In this ATM-dependent mechanism, γH2AX is specifically propagated throughout an entire topologically associated domain (TAD). TADs are structured chromatin domains actively maintained by cohesin and CCCTC-binding factor (CTCF) and formed by loop extrusion, in which chromatin is pushed through the cohesin molecules until opposing CTCF are encountered (Figure 4E and Rajarajan et al., 2016; Marchal et al., 2019). The authors propose that a DSB blocks extrusion, leading to unidirectional loop extrusion with the DSB repair machinery anchored one side of the extrusion process, allowing ATM to phosphorylate H2AX as the nucleosomes are extruded (Arnould et al., 2021) (Figure 3D). While γH2AX spreading within TADs may be facilitated by loop-extrusion, it is likely to be additive with phosphorylation via the previously described the MDC1/MRN/ATM positive feedback loop.

The phosphorylation of the TQXF motif of MDC1 by ATM provides a binding site for RNF8, an E3 ubiquitin ligase (Figures 4E, 5A and Nowsheen et al., 2018; Salguero et al., 2019). RNF8 interacts with several E2 enzymes, including the ubiquitin charged proteins UBCH8 and UBC13, leading to catalysis of either K48- or K63-linked ubiquitin chains, respectively (Lok et al., 2012). Ubiquitination is best known for marking proteins for degradation by the proteasome via K48-linked chains, but the K63-linkage plays has an important role as a signalling mark in the DSB response, as well as other pathways (e.g., protein kinase activation, and receptor endocytosis) (Lok et al., 2012). There has been some discussion on the major target of RNF8 in the DSB response. It was initially reported that RNF8 ubiquitinates histone H1 (Thorslund et al., 2015). However, more recent data established that RNF8 targets a protein termed Lethal(3)malignant brain tumour-like protein 2 (L3MBTL2) (Nowsheen et al., 2018). L3MBTL2 contains malignant brain tumour (MBT) repeats, which often function as ‘chromatin readers’ able to bind to histone modifications, and is one of at least three MBT-containing proteins active in the DSB response (Bonasio et al., 2010). Like RNF8, L3MBTL2 is also recruited to the MDC1 scaffold, this time by an ATM-dependent phosphorylation of L3MBTL2 (S335) which interacts with the MDC1 FHA domain (Figures 4E, 5A). The proximity of RNF8 and L3MBTL2, both bound to MDC1, facilitates polyubiquitination of L3BMTL2 (K659, via K63 linkages) by RNF8. This polyubiquitination serves as a platform for the binding of RNF168, another E3 ubiquitin ligase. The key role of RNF168 is mono-ubiquitination of H2A isoforms, including H2AX, on residues K13 and K15 [H2A(X)K13/15ub] (Mattiroli et al., 2012). Although RN168 can ubiquitinate both K13 and K15 residues, the role of K13ub in the DSB response is not understood; however, the damage-inducible ubiquitination of K15 is required for both 53BP1 and BRCA1 recruitment (Figure 5B and Mattiroli et al., 2012).

In addition to their transduction of the ATM damage signal, RNF8 and RNF168 also have other regulatory roles in the Early DSB response (Lok et al., 2012; Bartocci and Denchi, 2013). For example, the monoubiquitin on H2A(X)K13/15 can be extended by RNF8; while this polyubiquitination has unclear effects on 53BP1 and BARD1 binding, it is required for recruitment of RAP80 in a complex with BRCA1 (Hu et al., 2011). Other roles for RNF8 and RNF168 in the DSB response include ubiquitination-dependent regulation of L3MBTL1, KDM4A (JMJD2A), and 53BP1. However, these roles have not been fully elucidated and involve K48-linkages more typically involved in proteolysis. In addition, RNF8-dependent ubiquitination of NBS1 may aid the stabilization of MRN at DSBs (Lu et al., 2012). Furthermore, a poorly characterised scaffold protein, WRAP53β, has been reported to contribute to RNF8 recruitment through an unknown mechanism involving phosphorylation by ATM (at S64) and co-localisation with MDC1 (Henriksson et al., 2014; Rassoolzadeh et al., 2015; Coucoravas et al., 2017).

It is likely that there are many other undiscovered regulators of ATM recruitment and early phosphorylation events. A further example is the transcription factor SP1, which is phosphorylated by ATM and co-localises with γH2AX and members of the MRN complex, although its mechanism of interaction and regulatory impact have not yet been reported (Beishline et al., 2012). Finally, ufmylation of MRE11 on K282 has been reported to promote ATM activation, although the mechanistic details remain to be characterised (Wang et al., 2019). In fact, it is likely that many more details of how ATM regulates the response to DSBs remain to be reported and dissected and will add still further complexity to an already complex pathway. To date, the ‘major players’ required to transduce the Early DSB response include ATM, MRN, MDC1, RNF8, L3MBTL2, and RNF168, while multiple additional proteins are required to fine tune this signal transduction pathway.

## The Role of 53BP1 and BRCA1 in the Late Double Strand Break Response

**FIGURE 6:**
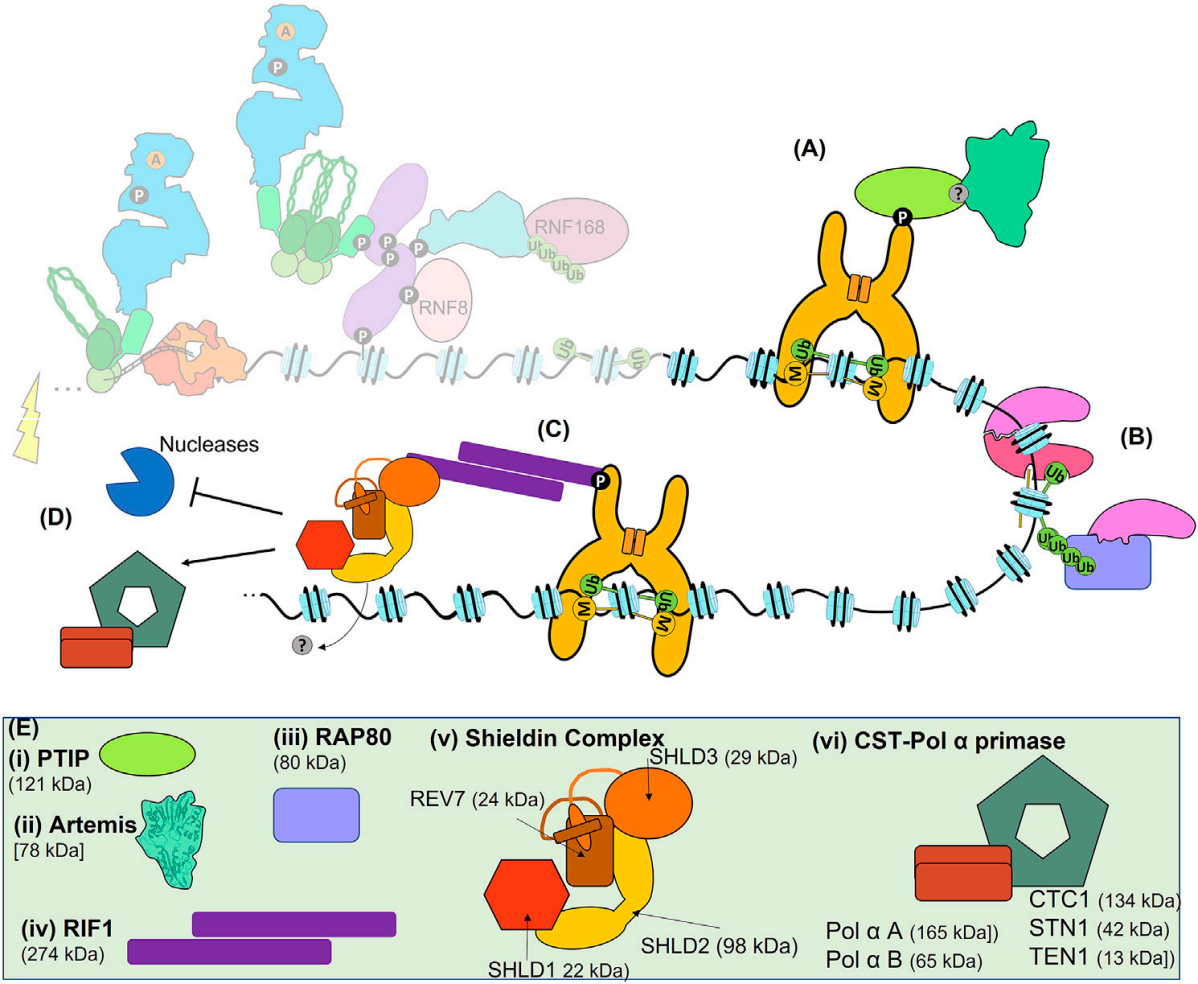
The Late response and pathway choice. **(A)** 53BP1-dependent recruitment of PTIP recruits Artemis to sites of DSBs for slow-kinetic cNHEJ. **(B)** Recruitment of BRCA1 to sites of DSBs can be BARD1-dependent, RAP80-dependent, MRN-dependent (not shown) or PARP1-dependent (not shown). **(C)** Recruitment of RIF1 and Shieldin to 53BP1. **(D)** 53BP1, RIF1, and Shieldin can block resection, or recruit the CST-Polα primase complex for gap fill-in, promoting the fidelity of both slow-kinetic cNHEJ and HR. **(E)** Schematics or structures of PTIP, Artemis, RAP80, RIF1, Sheildin and CST- CST-Polα primase. Where known structure is superimposed within overall architecture as illustrated. **(i)** PTIP is recruited to phosphorylated 53BP1. **(ii)** Artemis is the nuclease responsible for the 1–5 nt resection required for slow-kinetic cNHEJ. **(iii)** RAP80 can bind to polyubiquitination of H2A(X)K13 and H2A(X)K15 to recruit BRCA1. **(iv)** RIF1 forms a dimer via its large N-terminal domain, which can also bind directly to DNA (not shown), and interacts with 53BP1 via phosphorylation. **(v)** The Shieldin complex is made up of SHLD1, SHLD2, SHLD3, and REV7. REV7 and SHLD3 undergo conformational changes that facilitate their interaction, the so-called ‘seatbelt’ interaction. **(vi)** The CST complex forms a decameric supercomplex containing CTC1, STN1, and TEN1 (Lim et al., 2020) and interacts with Polα-primase. Polα-primase itself is composed of two subunits A and B.

Emerging data has demonstrated that two histone modifications are critical for pathway choice between NHEJ and HR (Fradet-Turcotte et al., 2013; Pellegrino et al., 2017; Nakamura et al., 2019; Becker et al., 2020; Dai et al., 2021; Hu et al., 2021; Morris, 2021). Ubiquitination of H2A isoforms [H2A(X)K13/15ub] together with the methylation state of histone H4 lysine 20 (either H4K20me0 or H4K20me2) recruit the critical readers of these bivalent chromatin marks, 53BP1 and BARD1, which is in complex with BRCA1 (Figure 5B). The control of these two PTMs is highly regulated; H2A(X)K13/15 is initially mono-ubiquitinated by the E3 ligase RNF168 in the chromatin flanking DNA damage, while the methylation of H4K20 is widespread throughout the genome. The recruitment of 53BP1 and BARD1-BRCA1 to these histone modifications occurs during the Late stage of the DSB response, and constitutes some of the last steps prior to pathway choice. Here we will briefly discuss the known mechanisms underlying the choice between slow-kinetic cNHEJ and HR (Figure 6 and reviewed in Panier and Boulton, 2013; Ceccaldi et al., 2016; Krenning et al., 2019; Scully et al., 2019; Mirman and de Lange, 2020; Ronato et al., 2020).

Previously we discussed the regulation of H2A ubiquitination [H2A(X)K13/15ub], which appears to be the critical damage-dependent regulatory event of the Early response. Di-methylation of H4 (H4K20me2) is largely constitutive and widely distributed throughout the genome. Importantly, for BARD1-BRCA1 recruitment, immediately post DNA replication, newly incorporated nucleosomes are transiently unmethylated (H4K20me0), although the existing nucleosomes retain methylation (H4K20me2) (Botuyan et al., 2006; Saredi et al., 2016; Nakamura et al., 2019; Becker et al., 2020). H4K20me2 is normally methylated by three methyltransferases, where SET8 (also termed SETD8, Pr-SET7, and KMT5A) provides the initial monomethylation, then SUV4-20H1 and its homologue SUV4-20H2 add the second and even a third methyl group (Jørgensen et al., 2013). Demethylation can occur via two RAD23 homologues, hHR23A and hHR23B (Cao et al., 2020). In addition to the post replication control of H4K20 methylation, H4K20me2 can be masked by either KDM4A (JMJD2A) or L3MBTL1 prior to damage (Acs et al., 2011; Butler et al., 2012; Mallette et al., 2012). Upon DNA damage, the concerted action of RNF8 and RNF168 ubiquitinate KDM4A via K48-linkage that targets it for proteasomal degradation, revealing the H4K20me2 mark for 53BP1 binding. Unmasking L3MBTL1 to reveal H4K20me2 is achieved somewhat differently. RNF8 and RNF168 are required to recruit the AAA-ATPases valosin-containing protein (VCP) and nuclear protein localization protein 4 (NPL4) to DSBs in order to remove L3MBTL1 freeing the H4K20me2 mark for 53BP1 (Jacquet et al., 2016).

In the context of a DSB, and the associated H2A ubiquitination [H2A(X)K15ub] of the flanking chromatin, the greatest binding affinity of 53BP1 and BARD1 is to H4K20me2 and H4K20me0, respectively (Pellegrino et al., 2017; Nakamura et al., 2019; Becker et al., 2020; Dai et al., 2021). Although H4K20me2 is abundant, it is also known to be damage-inducible via the histone methyltransferase MMSET (also known as NSD2 or WHSC1), which is recruited in a γH2AX- and MDC1-dependent manner (Pei et al., 2011). This could be particularly important for regions of the genome relatively depleted in the H4K20me2 modification. More typically, the H4K20me2 is only absent on newly synthesised chromatin. Thus, the brief availability of H4K20me0 in newly replicated chromatin facilitates recruitment of BARD1, immediately after replication fork passage, which directs repair towards HR as BARD1 forms a heterodimer with BRCA1 via the RING domain of BRCA1 (Figure 5C). BARD1 binds to H4K20me0 through its ankyrin repeat domain (ARD) domain, while its BRCT domain binds H2A(X)15ub (Nakamura et al., 2019). The affinity of BARD1 for H2A(X)K15ub is higher than that of 53BP1 (Dai et al., 2021). Once H4K20 becomes methylated, the window for repair via HR closes and repair is once more directed towards the slow-kinetic cNHEJ pathway. Although this is not the only method of BRCA1 recruitment, as BRCA1 forms many complexes, including BRCA1-A (Abraxas & RAP80 containing), BRCA1-B (BACH1 containing), BRCA1-C (CtIP and MRN containing) and the BRCA1/PALB2 complex, that are separately recruited (Figure 6B and reviewed in Her et al., 2016). As previously noted, it seems that BRCA1-A complex can be recruited to sites of DSBs by interaction between RAP80 and polyubiquitination of either H2A(X)K13 or H2A(X)K15, which is extended from monoubiquitination of either residue by RNF8 (Mattiroli et al., 2012). The post replication window during which newly incorporated histone H4 remains unmodified at lysine 20 methylation suggests a mechanism of how the cell successfully deals with one-ended DSBs that can occur at replication forks. One-ended DSBs cannot be accurately repaired by slow-kinetic cNHEJ (joining to another one-ended DSB elsewhere in the genome would result in a chromosomal rearrangement) and are instead repaired via break induced replication (BIR), a homology-dependent mechanism requiring the sister chromatid (Anand et al., 2013). While there are multiple mechanisms by which BRCA1 is recruited to two-ended DSBs, precisely how BRCA1 outcompetes 53BP1 to favour HR at those breaks preferentially repaired by this pathway remains to be fully elucidated.

The structure of 53BP1 allows it to bind to the bivalent damage-induced H2A(X)15ub and the largely constitutive H4K20me2 (see above and Fradet-Turcotte et al., 2013; Mirman and de Lange, 2020; Ronato et al., 2020). The C-terminus of 53BP1 consists of a tandem Tudor domain with a closely associated ubiquitin-dependent recruitment (UDR) motif, followed by a tandem BRCT domain which separately binds p53 (Figure 5C). The Tudor domain of 53BP1 specifically binds H4K20me2, while the UDR motif binds monoubiquitinated H2A(X)K15ub, but not H2A(X)K13ub (Botuyan et al., 2006; Panier et al., 2012; Uckelmann and Sixma, 2017). Effective binding of 53BP1 to these marks also requires its constitutive dimerization, achieved via its oligomerisation domain (OD), and facilitated by its interaction with DYNLL1 (also LC8). Interestingly, DYNLL1 interaction with MRE11 disrupts its nuclease activity, suggesting another mechanism by which 53BP1 inhibits resection (He et al., 2018). The large N-terminus of 53BP1 is unstructured and contains 28 S/TQ sites that can be phosphorylated by ATM and form a platform for recruitment of multiple factors such as RIF1, which in turn leads to the recruitment of Shieldin (Figure 6C and Findlay et al., 2018; Ghezraoui et al., 2018; Gupta et al., 2018).

The Shieldin complex, consisting of REV7 (MAD2L2), SHLD1, SHLD2, and SHLD3 is recruited to DSBs to block resection (Figure 6E). Shieldin can also recruit Polα-primase via its accessory factor CTC1-STN1-TEN1 (CST) to achieve the correct balance between resection and fill-in DNA synthesis (Figure 6D and Mirman et al., 2018). This may allow slow-kinetic cNHEJ to occur with higher fidelity and indicates an active role for 53BP1 in efficient slow-kinetic cNHEJ. A further active role for 53BP1 in slow-kinetic cNHEJ is suggested by its recruitment of PTIP, which in turn has functions in localising Artemis to DSBs that must be processed prior to repair (Figure 6A and Callen et al., 2013; Wang et al., 2014). The nuclease activity of Artemis is then required to process the DNA end (Riballo et al., 2004). Intriguingly, in addition to slow-kinetic cNHEJ, Artemis has also been shown to promote HR by removing lesions or secondary structures that inhibit repair by either pathway (Beucher et al., 2009). Regardless of these active roles in slow-kinetic cNHEJ, 53BP1-dependent recruitment of RIF1 and Shieldin inhibits HR, as well as the more mutagenic Alt-EJ, BIR, and SSA mechanisms of DSB repair.

53BP1 is clearly important for slow-kinetic cNHEJ and regulation of HR. These roles are supported by its recruitment into chromatin spanning megabases of DNA either side of a DSB, as well as its focal recruitment into large, micron sized, phase-separated condensates (Clouaire et al., 2018; Kilic et al., 2019). 53BP1 foci have been recently resolved into substructures termed ‘nanodomains’, which appear to correlate with TADs (Ochs et al., 2019; Caron and Polo, 2020). By mechanisms involving RIF1 and Shieldin, that have not been fully deciphered, these nanodomains reorganise into circular structures (termed ‘microdomains’). Each microdomain is composed of about five 53BP1 nanodomains/TADs, only one of which contains the DSB. The relationship between spherical foci and circular microdomains is unclear, but the arrangement of nanodomains might protect the integrity of chromatin in those TADs close to the TAD harbouring the DSB. Interestingly, pro-resection factors localises to the centre of the microdomain, likely to segregate such proteins away from the DNA until such time as they are needed, while anti-resection factors congregate within individual nanodomains.

Regulation of 53BP1 is even more complex than its histone modification-dependent recruitment. 53BP1 is recruited to DSBs that are ultimately repaired by either slow-kinetic cNHEJ or HR. As discussed, its role in slow-kinetic cNHEJ is not just limited to inhibition of resection but it may also have active roles in slow-kinetic cNHEJ (e.g., recruitment of Artemis, CST-Polα-primase), while in HR 53BP1 promotes fidelity by preventing excessive resection (Wang et al., 2014; Ochs et al., 2016; Löbrich and Jeggo, 2017; Mirman et al., 2018; Zhao et al., 2020; Kelich et al., 2021). Given such important roles, it is not surprising that 53BP1 recruitment is tightly regulated by multiple additional mechanisms: 1) the Tudor interacting repair regulator (TIRR) can bind the Tudor domain of 53BP1 to block its H4K20me2 binding (Drané et al., 2017; Dai et al., 2018; Wang et al., 2018); 2) acetylation within the 53BP1 UDR domain (K1626/K1628) by CREB-binding protein (CBP) disrupts 53BP1 binding to the nucleosome (Guo et al., 2018); 3) RNF169, an RNF168 paralogue, appears to be able to antagonise 53BP1, as well as RAP80-BRCA1, accumulation at DSBs through a mechanism that remains enigmatic (Chen et al., 2012; Panier et al., 2012; Poulsen et al., 2012; An et al., 2018); 4) phosphorylation of the damage dependent H2AK15 ubiquitin tag (UbT12p) itself also inhibits binding of 53BP1, but not HR factors (Walser et al., 2020); intriguingly, and in contrast to these negative regulatory mechanisms, 53BP1 binding can be positively regulated by the kinesin, KIF18B, in a mechanism requiring a direct interaction with the 53BP1 Tudor domain and the motor activity of KIF18B (Luessing et al., 2021).

The regulation of 53BP1 and BRCA1 recruitment to DSBs defines the Late response to DSBs that occur prior to repair by specific pathways, and is clearly complex and not yet fully understood. Emerging data demonstrates that both 53BP1 and BARD1-BRCA1 can bind to related bivalent histone marks, providing a DNA damage histone code. Both factors can bind to the chromatin flanking the same DSB. Details of how BRCA1 outcompetes 53BP1 at some DSBs are emerging. In particular, at one-ended DSBs produced at replication forks specific recruitment of BARD1-BRCA1 drives repair towards HR. Precisely how BRCA1 outcompetes 53BP1 at two-ended DSBs destined for repair via HR is as yet unknown. Furthermore, improved resolution of how 53BP1 and BRCA1 are physically segregated within three-dimensional space proximal to DSBs could provide important insight into pathway choice.

## The Role of KU70/80 and MRN and in the Late Response

Despite being two of the fastest-recruited proteins in the DSB response, KU70/80 and MRN have additional roles in repair choice that occur during the Early and Late response. Earlier we mentioned that proteins in the Immediate-Early damage response do not form visible foci, such as the ionising radiation induced foci (IRIF) that occur during the Early and Late response. While MRN foci have been well characterised, KU70/80 has also been shown to form detectable foci-formation at later timepoints (Britton et al., 2013). Focal recruitment indicates accumulation of sufficiently large amounts of protein that then become easy to detect by immunofluorescence. Unlike MRN foci, which can be visualised through conventional immunofluorescence, visualisation of KU70/80 foci requires pre-treatment with RNAseA and pre-extraction buffer (Britton et al., 2013). This is likely because, in addition to binding DNA ends, KU70/80 is also believed to bind RNA, also found in foci (Fijen and Rothenberg, 2021). Thus, revealing DSB-dependent foci during the ‘Late’ response appears to require removal of RNA to facilitate antibody access to KU70/80 (Britton et al., 2013; Sharma et al., 2021). However, neither KU70/80 nor MRN foci have been demonstrated within the Immediate-Early response, which takes place within the seconds immediately after DSBs formation and during which fast-kinetic cNHEJ occurs. Other than its DNA end binding activity, and possibly also a reported interaction between PARP1 and KU70/80, the mechanism of KU70/80 recruitment, particularly into foci, is less defined than for MRN, which is primarily ATM- and MDC1-dependent as previously discussed. Although, as a further complication, there is evidence that binding of human single-stranded DNA binding protein 1 (hSSB1) to resected DNA facilitates enhanced recruitment of MRN and increased MRE11 endonuclease activity (Richard et al., 2008; Richard et al., 2011a; Richard et al., 2011b).

Importantly, it appears that while KU70/80 must be retained at DSBs for slow-kinetic cNHEJ, for HR KU70/80 has to be evicted during the Late response. KU70/80 eviction is achieved by a combination of nucleolytic and proteolytic activities. Interestingly, the major nuclease implicated in KU70/80 eviction is MRE11, indicating crosstalk between these two end binding complexes, while CtIP also plays a role (Langerak et al., 2011; Chanut et al., 2016; Myler et al., 2017). Proteolytic eviction of KU70/80 is regulated by RNF8 and RNF138, yet another E3 ubiquitin ligase, which can tag KU70/80 for degradation using K48-linked polyubiquitin (Feng and Chen, 2012; Ismail et al., 2015). Ubiquitination of KU70/80 appears to be promoted by yet another post translational modification, neddylation (Brown et al., 2015).

It is possible that MRN could also contribute to the proximal ‘melting’ of the broken DNA ends to facilitate the loading of RNA polymerases. The resulting non-coding RNAs (ncRNAs) have been reported to be processed by the RNAses DROSHA and DICER to become the so-called DNA damage response or damage-inducible RNAs (DDRNA or diRNA), reported to regulate the DSB response (Francia et al., 2012; Wei et al., 2012). Another recently reported role for RNA in the DSB response is to hybridise to the 3′ overhanging strand after resection thereby protecting it from nucleases. RNA Pol III has been reported to synthesise the RNA that forms these transient RNA-DNA hybrids, and impacts upon high fidelity repair by both slow-kinetic cNHEJ and HR (Liu et al., 2021). Indeed, beyond the scope of this review, there is emerging evidence that RNA plays many important roles in the DSB response (Chowdhury et al., 2013; Barroso et al., 2019; Crossley et al., 2019; Bader et al., 2020; Ketley and Gullerova, 2020; Guiducci and Stojic, 2021; Jimeno et al., 2021; Marnef and Legube, 2021; Palancade and Rothstein, 2021). Perhaps the ability of KU70/80 to bind RNA could be important with respect to the emerging roles for RNA in the responses to DSBs.

## Downstream Double Strand Break Repair Pathways

After the Late DSB response, largely constituting a delicate balance between 53BP1 and BRCA1 recruitment, the remaining DSBs can be repaired by either slow-kinetic cNHEJ or HR (Figures 1E,I). However, these two high-fidelity repair pathways are often counted among five distinct pathways. These are: i) slow-kinetic cNHEJ (note that fast-kinetic cNHEJ is an Immediate-Early response, see Figure 2); ii) alternative end joining (Alt-EJ; often referred to as microhomology-mediated EJ, or MMEJ); iii) break induced replication (BIR); iv) single strand annealing (SSA), and v) gene conversion (GC), which can result from two distinct HR mechanisms, either synthesis dependent strand annealing (SDSA, also called short tract GC) or double Holliday Junctions (dHJ, also called long tract GC) mediated recombination (Figures 1E–I and Mehta and Haber, 2014; Chang et al., 2017; Malkova, 2018; Krenning et al., 2019). How the cellular DSB repair machineries funnel DSBs into the possible repair outcomes is not yet fully understood, but influencing factors include cell cycle stage, chromatin context (especially with respect to transcriptional status), the type and extent of the breaks, and the amount of resected ssDNA (Ronato et al., 2020). The historical perspective that cNHEJ and HR are resection independent or dependent, respectively, has been revised by the realisation that in addition to Alt-EJ, some slow-kinetic cNHEJ also relies upon resection (Shibata and Jeggo, 2019).

Slow-kinetic cNHEJ accounts for repair of about 20% of IR induced DSBs, and has been termed ATM, 53BP1-, or Artemis-dependent cNHEJ and, as it requires some limited (1–5 nt) resection, is additionally termed resection-dependent cNHEJ (Figure 1E and Chang et al., 2017; Jeggo and Löbrich, 2017). This pathway also depends upon other indirect factors impacting upon pathway choice, including RIF1, Shieldin, and CST-Polα primase. Whereas the core cNHEJ proteins are required by both fast- and slow-kinetic cNHEJ, 53BP1, RIF1, and Shieldin are anti-resection factors and CST- Polα primase balances resection with *de novo* DNA synthesis, likely improving fidelity (Mirman et al., 2018). However, fast- and slow-kinetic cNHEJ differ in their ability to repair simple versus complex DSBs, have different recruitment pathways, and are used to different extents throughout the cell cycle (Setiaputra and Durocher, 2019; Shibata and Jeggo, 2020a; Shibata and Jeggo, 2020b; Qi et al., 2021). Intriguingly, emerging data suggests that slow-kinetic cNHEJ can avoid mutagenic deletions by using RNA molecules as homology templates for retrieving sequence information that can be lost during resection (Storici et al., 2007; Chakraborty et al., 2016; Meers et al., 2016; Mazina et al., 2017).

Alt-EJ encompasses vestigial NHEJ repair pathways that do not require KU70/80, XRCC4, or LIG4 (Figure 1F and Iliakis et al., 2015; Wyatt et al., 2016; Dutta et al., 2017; Hanscom and McVey, 2020; Ramsden et al., 2021). These repair subpathways occur after Artemis and CtIP-dependent slow-kinetic cNHEJ fails to repair the DSB, and when there is insufficient homology (less than 25 nt) for HR. Interestingly, Alt-EJ pathways can still occur in cells with functioning cNHEJ and HR, albeit at a frequency of just 0.5%–1% (Hanscom and McVey, 2020). Given the many descriptors (a-EJ, alterative NHEJ, backup NHEJ, MMEJ (microhomology-mediated end joining), TMEJ [polymerase theta (Pol θ)-Mediated End joining], Synthesis-dependent MMEJ, etc.) and the obvious confusion generated, Alt-EJ subpathways may best be considered as being either Pol θ−dependent or independent. During, MMEJ resection reveals microhomologies allowing annealing, followed by removal of the 3′ non-homologous tails, gap filling, and ligation. It is interesting to note that PARP1 plays a role in Alt-EJ subpathways such as MMEJ (Mansour et al., 2010; Dutta et al., 2017). TMEJ, on the other hand, still uses microhomologies, but also relies on Pol θ in order to prime the synthesis of up to 25 nt of nascent DNA (Hanscom and McVey, 2020). More recently, TMEJ has been shown to be cell cycle regulated, repairing one-ended DSBs that arise in S-phase in early mitosis (Llorens-Agost et al., 2021). In this pathway, RAD52 and BRCA2 delay TMEJ until early mitosis, by which time one-ended DSBs have been converted to two-ended DSBs. A recent study also demonstrated that the polymerase activity of Pol θ is not the only function required for TMEJ; amazingly, its DNA polymerisation domain can also function nucleolytically for 3′ end trimming (Zahn et al., 2021). When we consider NHEJ as a whole network of pathways, it is important to remember that fast-kinetic cNHEJ occurs upstream within the Immediate-Early response, while all other subdivisions, including slow-kinetic cNHEJ, MMEJ, TMEJ, and any other Alt-EJ pathways, are all resection-dependent repair pathways.

In mitotic cells HR has three main subdivisions: GC, BIR, and SSA (Jackson, 2002; Mehta and Haber, 2014; Chang et al., 2017; Krenning et al., 2019; Pham et al., 2021). GC is the highest-fidelity repair; in yeast requiring just 20–80 nt of *in trans* homology, and resecting 2–6 kb, while in mammalian cells the minimal *in trans* homology is unclear, but resection can occur for up to 3.5 kb (Ronato et al., 2020). In mammals, GC is dependent upon the nuclease activity of MRN and other proteins such as CtIP, BLM, EXO1, RPA1, BRCA1, PALB2, BRCA2, XRCC3, RAD51, and RAD54 (Figure 1I). This repair pathway requires end resection, ssDNA protection, search for homology, strand invasion, and resolution of the resulting Holliday Junction (Jackson, 2002; Li and Heyer, 2008). GC has two subdivisions: SDSA and dHJ mediated recombination, which are also referred to as short tract GC (STGC) and long tract GC (LTGC), respectively (Elbakry and Löbrich, 2021). In SDSA, an unstable displacement loop (D-loop) is formed as an intermediate composed of a double stranded DNA double helix invaded by the broken DNA end, leading to short-tract DNA synthesis. The second end of the break is then annealed to this newly synthesised DNA, resulting in repair that is cross-over independent. This is the most common form of DSB repair, as it minimises the chance of mutations to DNA near the DSB (Pham et al., 2021). On the other hand, dHJ resolution begins with the invasion of the broken strand to form a stable D-loop, followed by long-tract DNA synthesis. The second end of the DSB is eventually captured, leading to the formation of joint molecules. The resolution of these joint molecules results in cross over and non-cross over events with equal frequencies (Elbakry and Löbrich, 2021).

It is interesting to note that the involvement of BRCA1 and BRCA2 in HR is an area of intensive research stimulated by the roles of these DSB repair factors in heritable *BRCA* defective breast and ovarian cancers. Furthermore, *BRCA* defective cancer cells are sensitive to PARP inhibition (Antolin et al., 2020; Jannetti et al., 2020; Rose et al., 2020), and this synthetic lethality suggests that PARP and BRCA1/2 function in different pathways. The mechanism by which PARP inhibitors function remains to be fully deciphered and is subject to much debate, but it has been proposed to be due to defective SSB repair, which results in one-ended DSBs during S phase that require HR for their repair (Helleday, 2011; Murai et al., 2012; Horton et al., 2014). However, it is likely that Artemis-dependent, or slow-kinetic cNHEJ, also contributes to PARP inhibition-dependent lethality in HR-defective cells (Patel et al., 2011; De Lorenzo et al., 2013), consistent with PARP performing some roles in multiple DSB repair pathways.

BIR is a sub-pathway of HR which uses the invading strand for long-range DNA synthesis without the engagement of a second DSB end (Elbakry and Löbrich, 2021). It therefore repairs one-ended DSBs arising from fork collapse and provides an alternative mechanism for telomere maintenance when telomerase is lost (Figure 1G and Malkova, 2018). In budding yeast, BIR requires approximately 72 nt of homology and can resect up to 1 kb (Ronato et al., 2020). This recombination-based method of conservative DNA replication copies from a template DNA until the end of the DNA template. The invasion of the single DNA end and subsequent replication during BIR relies on RPA, Rad52, Rad51, and to some extent, Rad54, Rad55, and Rad59 (Anand et al., 2013; Malkova, 2018). Although it is not currently known what restrains BIR at two ended breaks and promotes GC, the proteins Rad52, Rad58, Mph1, and MRX have been implicated in yeast studies (Pham et al., 2021).

SSA is not dependent on a sister chromatid for homology and results in deletions (Figure 1H). Resection reveals *in cis* homologous repeat sequences which then anneal together with the resulting 3′ flap structures being removed (Figure 1H and Onaka et al., 2020). Studies in budding yeast have shown that SSA relies on 63–89 bp homology, while the end is resected until homology occurs (Ronato et al., 2020). In yeast or mammalian cells, mutagenic SSA occurs when GC is unavailable, for example, when RAD51 or RAD54 are depleted, the cell switches to the RAD52-dependent SSA repair (Ochs et al., 2016; Onaka et al., 2020).

When considering the DDR, there are other pathways that tie into these described repair pathways that have not been discussed in this review, for example, DSBs arising at a replication fork. The kinase ATR can be activated in response to resected DSBs, but is most often activated in response to the elevated levels of ssDNA couated with RPA, that occurs at stalled replication forks. Such structures can be converted into DSBs by nucleolytic attack or fork collapse (Burger et al., 2019). Alternately, ATR can be activated if repair is unsuccessful, as it is involved in checkpoint signalling and cell fate. Additionally, ICL repair is a critical pathway that repairs one of the most complex DNA lesions (Scully et al., 2019; Panday et al., 2021; Semlow and Walter, 2021). Because ICL repair generates a transient DSB as an intermediate, that is protected within the context of ICL repair, it should be included in the discussion of DSB repair pathways. It depends on FA core proteins, as well as downstream repair proteins involved in both HR and cNHEJ. The repair at an ICL consists of an unhooking step, *trans* lesion synthesis, excision repair, strand invasion, and resolution. During S-phase, there are complex repair requirements at single or converging forks, while replication-independent ICL repair can also outside of S-phase (Semlow and Walter, 2021). Processing of the DSB after the unhooking step depends upon HR proteins for repair via strand invasion and resolution. It should be noted that the DSB produced during ICL repair is protected within the context of this repair pathway. It is therefore not likely to be sensed as a classic DSB that activates the Immediate-Early and Early response.

It is important to note that cNHEJ, both fast- and slow-kinetic, as well as HR appear to be the default pathways in healthy wild-type mammalian cells and they are not usually error prone as they have evolved to operate with high fidelity (Ceccaldi et al., 2016). The physiological relevance of the alternate repair pathways Alt-EJ, SSA, and BIR under normal conditions remains an open question. These mutagenic pathways occur in the absence of certain cNHEJ and HR factors or upon non-physiological levels of replication stress, for example in cancerous cells. Under such cellular conditions, elevated levels of error-prone DSB repair may therefore reflect the enzymatic capabilities of the remaining proteins (Khanna and Jackson, 2001; Iliakis et al., 2019).

## Concluding Remarks and Future Directions

Here, we have presented an integrated view of the pre-repair DSB response at its three main stages, Immediate-Early, Early, and Late. Although there is no clear consensus on a precise DSB sensor in the field, the Immediate-Early response consists of the initial DSB sensing and signalling that occurs within seconds of DSB formation. While PARPs have well defined roles in SSB repair, there is emerging data implicating some roles for PARP1, PARP2, and PARP3 upstream of DSB repair. Furthermore, the complex recruitment and interplay between PARP, KU70/80, and MRN contributes to downstream pathway choice. In addition, we support emerging evidence for fast-kinetic cNHEJ responsible for the rapid repair of most DSBs during the Immediate-Early response. The remaining breaks require processing before repair. The activation of ATM and the associated chromatin dynamics constitutes the Early response. This culminates in damage-dependent ubiquitination events permissive for recruitment of Late response proteins, such as the 53BP1 and BRCA1 scaffold proteins.

Every step of the pre-repair responses, Immediate-Early, Early, and Late, appears to be important for pathway choice. This requires complex integration of multiple factors to achieve the optimal outcome, which in turn will be specific to the context of each DNA lesion. These factors include the complexity of the DSB itself, chromatin context, cell cycle phase, and availability of the specific repair factors required to achieve the highest fidelity possible. Critical molecular events include the PARP-dependent PARylation response, the recruitment of KU70/80 and/or MRN, dynamic chromatin decondensation and condensation, the activation of ATM, and damage-dependent histone modifications defining a histone code for DSB repair. While there is crosstalk between the Immediate-Early, Early, and Late responses, according to our current understanding it is not until after 53BP1 and BRCA1 recruitment that a cell commits to a specific DSB repair pathway*.* However, much remains to be discovered about how these responses crosstalk, overlap, and compete.

A key emerging question is apparently simple, yet of deep complexity: for those breaks that are not immediately ligated, at what stage is a DSB committed to a specific repair pathway? Instead of pathway choice occurring downstream of 53BP1 and BRCA1, could it not be more useful to consider pathway choice as a continuous process? It is likely that regulation and crosstalk between the pre-repair pathways allows integration of the many factors required for normal maintenance of genome stability. A related question is whether, if repair fails, can the repair machinery backtrack and attempt to repair the lesion using an alternative high-fidelity approach, before resorting to a more error-prone mechanism. Additionally, the interplay between the PARP-dependent Immediate-Early response and the ATM- and chromatin-dependent Early response has not been fully deciphered. Also, despite its pleiotropic roles throughout the DSB response, how CK2 is activated to specifically regulate so many steps remains enigmatic. It is important to consider that highly error prone mechanisms are unlikely to be physiologically relevant under normal conditions, and are likely to be rare events in normally growing unstressed wild-type cells. Under suboptimal conditions, such as the loss of specific DSB factors that occurs during cancer, or where elevated and non-physiological levels of damage are induce by exogenous agents, repair outcomes become skewed towards mutation. Under such conditions, and if apoptosis is not triggered, repair is likely to proceed using whatever machinery is available. Full understanding of the DSB response remains a challenge for the future. No doubt, these challenges will be met and will expand our evolving understanding of how Immediate-Early, Early, and Late DSB responses are coordinated and integrated to achieve the optimal downstream repair outcomes.

## References

[B1] Aberle L. Krüger A. Reber J. M. Lippmann M. Hufnagel M. Schmalz M. (2020). PARP1 Catalytic Variants Reveal Branching and Chain Length-specific Functions of poly(ADP-Ribose) in Cellular Physiology and Stress Response. Nucleic Acids Res. 48, 10015–10033. 10.1093/nar/gkaa590 32667640 PMC7544232

[B2] Acs K. Luijsterburg M. S. Ackermann L. Salomons F. A. Hoppe T. Dantuma N. P. (2011). The AAA-ATPase VCP/p97 Promotes 53BP1 Recruitment by Removing L3MBTL1 from DNA Double-Strand Breaks. Nat. Struct. Mol. Biol. 18, 1345–1350. 10.1038/nsmb.2188 22120668

[B3] Adamowicz M. Hailstone R. Demin A. A. Komulainen E. Hanzlikova H. Brazina J. (2021). XRCC1 Protects Transcription from Toxic PARP1 Activity during DNA Base Excision Repair. Nat. Cel Biol. 23, 1287–1298. 10.1038/s41556-021-00792-w PMC8683375 34811483

[B4] Aguilar-Quesada R. Muñoz-Gámez J. A. Martín-Oliva D. Peralta A. Valenzuela M. T. Matínez-Romero R. (2007). Interaction between ATM and PARP-1 in Response to DNA Damage and Sensitization of ATM Deficient Cells through PARP Inhibition. BMC Mol. Biol 8, 1. 10.1186/1471-2199-8-29 17459151 PMC1868035

[B5] Ali A. A. E. Timinszky G. Arribas-Bosacoma R. Kozlowski M. Hassa P. O. Hassler M. (2012). The Zinc-finger Domains of PARP1 Cooperate to Recognize DNA Strand Breaks. Nat. Struct. Mol. Biol. 19, 685–692. 10.1038/NSMB.2335 22683995 PMC4826610

[B6] An L. Dong C. Li J. Chen J. Yuan J. Huang J. (2018). RNF169 Limits 53BP1 Deposition at DSBs to Stimulate Single-Strand Annealing Repair. Proc. Natl. Acad. Sci. USA 115, E8286–E8295. 10.1073/PNAS.1804823115 30104380 PMC6126738

[B7] Anand R. P. Lovett S. T. Haber J. E. (2013). Break-induced DNA Replication. Cold Spring Harbor Perspect. Biol. 5, a010397. 10.1101/cshperspect.a010397 PMC3839615 23881940

[B8] Antolin A. A. Ameratunga M. Banerji U. Clarke P. A. Workman P. Al-Lazikani B. (2020). The Kinase Polypharmacology Landscape of Clinical PARP Inhibitors. Sci. Rep. 10, 1–14. 10.1038/s41598-020-59074-4 32066817 PMC7026418

[B9] Arnould C. Rocher V. Finoux A.-L. Clouaire T. Li K. Zhou F. (2021). Loop Extrusion as a Mechanism for Formation of DNA Damage Repair Foci. Nature 590, 660–665. 10.1038/s41586-021-03193-z 33597753 PMC7116834

[B10] Aymard F. Bugler B. Schmidt C. K. Guillou E. Caron P. Briois S. (2014). Transcriptionally Active Chromatin Recruits Homologous Recombination at DNA Double-Strand Breaks. Nat. Struct. Mol. Biol. 21, 366–374. 10.1038/nsmb.2796 24658350 PMC4300393

[B11] Ayoub N. Jeyasekharan A. D. Bernal J. A. Venkitaraman A. R. (2008). HP1-β Mobilization Promotes Chromatin Changes that Initiate the DNA Damage Response. Nature 453, 682–686. 10.1038/nature06875 18438399

[B12] Bader A. S. Hawley B. R. Wilczynska A. Bushell M. (2020). The Roles of RNA in DNA Double-Strand Break Repair. Br. J. Cancer 122, 613–623. 10.1038/s41416-019-0624-1 31894141 PMC7054366

[B13] Bakkenist C. J. Kastan M. B. (2015). Chromatin Perturbations during the DNA Damage Response in Higher Eukaryotes. DNA Repair 36, 8–12. 10.1016/J.DNAREP.2015.09.002 26391293 PMC4727245

[B14] Bakkenist C. J. Kastan M. B. (2003). DNA Damage Activates ATM through Intermolecular Autophosphorylation and Dimer Dissociation. Nature 421, 499–506. 10.1038/nature01368 12556884

[B15] Barroso S. Herrera‐Moyano E. Muñoz S. García‐Rubio M. Gómez‐González B. Aguilera A. (2019). The DNA Damage Response Acts as a Safeguard against Harmful DNA-RNA Hybrids of Different Origins. EMBO Rep. 20, e47250. 10.15252/embr.201847250 31338941 PMC6726908

[B16] Bartocci C. Denchi E. L. (2013). Put a RING on it: Regulation and Inhibition of RNF8 and RNF168 RING finger E3 Ligases at DNA Damage Sites. Front. Genet. 4. 10.3389/fgene.2013.00128 PMC3705210 23847653

[B17] Beck C. Boehler C. Guirouilh Barbat J. Bonnet M.-E. Illuzzi G. Ronde P. (2014). PARP3 Affects the Relative Contribution of Homologous Recombination and Nonhomologous End-Joining Pathways. Nucleic Acids Res. 42, 5616–5632. 10.1093/NAR/GKU174 24598253 PMC4027158

[B18] Becker J. R. Bonnet C. Clifford G. Groth A. Wilson M. D. Chapman J. R. (2020). BARD1 Links Histone H2A Lysine-15 Ubiquitination to Initiation of BRCA1-dependent Homologous Recombination. bioRxiv. 10.1101/2020.06.01.127951 PMC7619424 34321663

[B19] Becker J. S. Nicetto D. Zaret K. S. (2016). H3K9me3-Dependent Heterochromatin: Barrier to Cell Fate Changes. Trends Genet. 32, 29–41. 10.1016/j.tig.2015.11.001 26675384 PMC4698194

[B20] Beishline K. Kelly C. M. Olofsson B. A. Koduri S. Emrich J. Greenberg R. A. (2012). Sp1 Facilitates DNA Double-Strand Break Repair through a Nontranscriptional Mechanism. Mol. Cel. Biol. 32, 3790–3799. 10.1128/mcb.00049-12 PMC3430196 22826432

[B21] Benjamin R. C. Gill D. M. (1980). ADP-ribosylation in Mammalian Cell Ghosts. Dependence of poly(ADP-Ribose) Synthesis on Strand Breakage in DNA. J. Biol. Chem. 255, 10493–10501. 10.1016/S0021-9258(19)70490-6 7430132

[B268] Bensimon A. Schmidt A. Ziv Y. Elkon R. Wang S. Y. Chen D. J. (2010). ATM-Dependent And -Independent Dynamics Of The Nuclear Phosphoproteome After DNA Damage. Sci. Signal. 3. 10.1126/scisignal.2001034 21139141

[B22] Beucher A. Birraux J. Tchouandong L. Barton O. Shibata A. Conrad S. (2009). ATM and Artemis Promote Homologous Recombination of Radiation-Induced DNA Double-Strand Breaks in G2. EMBO J. 28, 3413–3427. 10.1038/EMBOJ.2009.276 19779458 PMC2752027

[B23] Biehs R. Steinlage M. Barton O. Juhász S. Künzel J. Spies J. (2017). DNA Double-Strand Break Resection Occurs during Non-homologous End Joining in G1 but Is Distinct from Resection during Homologous Recombination. Mol. Cel 65, 671–684. 10.1016/j.molcel.2016.12.016 PMC5316416 28132842

[B24] Bonasio R. Lecona E. Reinberg D. (2010). MBT Domain Proteins in Development and Disease. Semin. Cel Dev. Biol. 21, 221–230. 10.1016/J.SEMCDB.2009.09.010 PMC3772645 19778625

[B25] Botuyan M. V. Lee J. Ward I. M. Kim J.-E. Thompson J. R. Chen J. (2006). Structural Basis for the Methylation State-specific Recognition of Histone H4-K20 by 53BP1 and Crb2 in DNA Repair. Cell 127, 1361–1373. 10.1016/j.cell.2006.10.043 17190600 PMC1804291

[B26] Breslin C. Hornyak P. Ridley A. Rulten S. L. Hanzlikova H. Oliver A. W. (2015). The XRCC1 Phosphate-Binding Pocket Binds Poly (ADP-Ribose) and Is Required for XRCC1 Function. Nucleic Acids Res. 43, 6934–6944. 10.1093/NAR/GKV623 26130715 PMC4538820

[B27] Britton S. Coates J. Jackson S. P. (2013). A New Method for High-Resolution Imaging of Ku Foci to Decipher Mechanisms of DNA Double-Strand Break Repair. J. Cel Biol. 202, 579–595. 10.1083/JCB.201303073 PMC3734090 23897892

[B28] Brown J. S. Jackson S. P. (2015). Ubiquitylation, Neddylation and the DNA Damage Response. Open Biol. 5, 150018. 10.1098/rsob.150018 25833379 PMC4422126

[B29] Brown J. S. Lukashchuk N. Sczaniecka-Clift M. Britton S. le Sage C. Calsou P. (2015). Neddylation Promotes Ubiquitylation and Release of Ku from DNA-Damage Sites. Cel Rep. 11, 704–714. 10.1016/J.CELREP.2015.03.058 PMC4431666 25921528

[B30] Bryant H. E. Schultz N. Thomas H. D. Parker K. M. Flower D. Lopez E. (2005). Specific Killing of BRCA2-Deficient Tumours with Inhibitors of poly(ADP-Ribose) Polymerase. Nature 434, 913–917. 10.1038/nature03443 15829966

[B31] Burger K. Ketley R. F. Gullerova M. (2019). Beyond the Trinity of ATM, ATR, and DNA-PK: Multiple Kinases Shape the DNA Damage Response in Concert with RNA Metabolism. Front. Mol. Biosci. 6, 61. 10.3389/fmolb.2019.00061 31428617 PMC6688092

[B32] Butler L. R. Densham R. M. Jia J. Garvin A. J. Stone H. R. Shah V. (2012). The Proteasomal De-ubiquitinating Enzyme POH1 Promotes the Double-Strand DNA Break Response. EMBO J. 31, 3918–3934. 10.1038/emboj.2012.232 22909820 PMC3463844

[B33] Caldecott K. W. (2014a). DNA Single-Strand Break Repair. Exp. Cel Res. 329, 2–8. 10.1016/J.YEXCR.2014.08.027 25176342

[B34] Caldecott K. W. (2003). DNA Single-Strand Break Repair and Spinocerebellar Ataxia. Cell 112, 7–10. 10.1016/S0092-8674(02)01247-3 12526788

[B35] Caldecott K. W. (2014b). Protein ADP-Ribosylation and the Cellular Response to DNA Strand Breaks. DNA Repair 19, 108–113. 10.1016/J.DNAREP.2014.03.021 24755000

[B36] Callen E. Di Virgilio M. Kruhlak M. J. Nieto-Soler M. Wong N. Chen H.-T. (2013). 53BP1 Mediates Productive and Mutagenic DNA Repair through Distinct Phosphoprotein Interactions. Cell 153, 1266–1280. 10.1016/j.cell.2013.05.023 23727112 PMC3713552

[B37] Cao X. Chen Y. Wu B. Wang X. Xue H. Yu L. (2020). Histone H4K20 Demethylation by Two hHR23 Proteins. Cel Rep. 30, 4152–4164. 10.1016/j.celrep.2020.03.001 32209475

[B38] Caron M.-C. Sharma A. K. O’Sullivan J. Myler L. R. Ferreira M. T. Rodrigue A. (2019). Poly(ADP-ribose) Polymerase-1 Antagonizes DNA Resection at Double-Strand Breaks. Nat. Commun. 10. 10.1038/s41467-019-10741-9 PMC6609622 31273204

[B39] Caron P. Choudjaye J. Clouaire T. Bugler B. Daburon V. Aguirrebengoa M. (2015). Non-redundant Functions of ATM and DNA-PKcs in Response to DNA Double-Strand Breaks. Cel Rep. 13, 1598–1609. 10.1016/J.CELREP.2015.10.024 PMC4670905 26586426

[B40] Caron P. Polo S. E. (2020). Reshaping Chromatin Architecture Around DNA Breaks. Trends Biochem. Sci. 45, 177–179. 10.1016/j.tibs.2019.12.001 31882194

[B41] Casari E. Rinaldi C. Marsella A. Gnugnoli M. Colombo C. V. Bonetti D. (2019). Processing of DNA Double-Strand Breaks by the MRX Complex in a Chromatin Context. Front. Mol. Biosci. 6. 10.3389/fmolb.2019.00043 PMC6567933 31231660

[B42] Ceccaldi R. Rondinelli B. D’Andrea A. D. (2016). Repair Pathway Choices and Consequences at the Double-Strand Break. Trends Cel Biol. 26, 52–64. 10.1016/J.TCB.2015.07.009 PMC4862604 26437586

[B43] Chakraborty A. Tapryal N. Venkova T. Horikoshi N. Pandita R. K. Sarker A. H. (2016). Classical Non-homologous End-Joining Pathway Utilizes Nascent RNA for Error-free Double-Strand Break Repair of Transcribed Genes. Nat. Commun. 7. 10.1038/NCOMMS13049 PMC5059474 27703167

[B44] Chambon P. Weill J. D. Mandel P. (1963). Nicotinamide Mononucleotide Activation of a New DNA-dependent Polyadenylic Acid Synthesizing Nuclear Enzyme. Biochem. Biophysical Res. Commun. 11, 39–43. 10.1016/0006-291X(63)90024-X 14019961

[B45] Chang H. H. Y. Pannunzio N. R. Adachi N. Lieber M. R. (2017). Non-homologous DNA End Joining and Alternative Pathways to Double-Strand Break Repair. Nat. Rev. Mol. Cel Biol. 18, 495–506. 10.1038/nrm.2017.48 PMC7062608 28512351

[B46] Chanut P. Britton S. Coates J. Jackson S. P. Calsou P. (2016). Coordinated Nuclease Activities Counteract Ku at Single-Ended DNA Double-Strand Breaks. Nat. Commun. 7. 10.1038/ncomms12889 PMC5031800 27641979

[B47] Chapman J. R. Jackson S. P. (2008). Phospho‐dependent Interactions between NBS1 and MDC1 Mediate Chromatin Retention of the MRN Complex at Sites of DNA Damage. EMBO Rep. 9, 795–801. 10.1038/EMBOR.2008.103 18583988 PMC2442910

[B48] Chen J. Feng W. Jiang J. Deng Y. Huen M. S. Y. (2012). Ring finger Protein RNF169 Antagonizes the Ubiquitin-dependent Signaling cascade at Sites of DNA Damage. J. Biol. Chem. 287, 27715–27722. 10.1074/jbc.M112.373530 22733822 PMC3431699

[B49] Chowdhury D. Choi Y. E. Brault M. E. (2013). Charity Begins at home: Non-coding RNA Functions in DNA Repair. Nat. Rev. Mol. Cel Biol. 14, 181–189. 10.1038/nrm3523 PMC3904369 23385724

[B50] Clouaire T. Legube G. (2019). A Snapshot on the Cis Chromatin Response to DNA Double-Strand Breaks. Trends Genet. 35, 330–345. 10.1016/j.tig.2019.02.003 30898334

[B51] Clouaire T. Rocher V. Lashgari A. Arnould C. Aguirrebengoa M. Biernacka A. (2018). Comprehensive Mapping of Histone Modifications at DNA Double-Strand Breaks Deciphers Repair Pathway Chromatin Signatures. Mol. Cel 72, 250–262. 10.1016/j.molcel.2018.08.020 PMC6202423 30270107

[B52] Coucoravas C. Dhanjal S. Henriksson S. Böhm S. Farnebo M. (2017). Phosphorylation of the Cajal Body Protein WRAP53β by ATM Promotes its Involvement in the DNA Damage Response. RNA Biol. 14, 804–813. 10.1080/15476286.2016.1243647 27715493 PMC5519231

[B53] Couto C. A.-M. Wang H.-Y. Green J. C. A. Kiely R. Siddaway R. Borer C. (2011). PARP Regulates Nonhomologous End Joining through Retention of Ku at Double-Strand Breaks. J. Cel Biol. 194, 367–375. 10.1083/JCB.201012132 PMC3153639 21807880

[B54] Crossley M. P. Bocek M. Cimprich K. A. (2019). R-loops as Cellular Regulators and Genomic Threats. Mol. Cel 73, 398–411. 10.1016/j.molcel.2019.01.024 PMC6402819 30735654

[B55] Dai L. Dai Y. Han J. Huang Y. Wang L. Huang J. (2021). Structural Insight into BRCA1-BARD1 Complex Recruitment to Damaged Chromatin. Mol. Cel 81, 2765–2777. 10.1016/J.MOLCEL.2021.05.010 34102105

[B56] Dai Y. Zhang A. Shan S. Gong Z. Zhou Z. (2018). Structural Basis for Recognition of 53BP1 Tandem Tudor Domain by TIRR. Nat. Commun. 9. 10.1038/s41467-018-04557-2 PMC5974088 29844495

[B57] Daugaard M. Baude A. Fugger K. Povlsen L. K. Beck H. Sørensen C. S. (2012). LEDGF (P75) Promotes DNA-End Resection and Homologous Recombination. Nat. Struct. Mol. Biol. 19, 803–810. 10.1038/nsmb.2314 22773103

[B58] De Jager M. Van Noort J. Van Gent D. C. Dekker C. Kanaar R. Wyman C. (2001). Human Rad50/Mre11 Is a Flexible Complex that Can Tether DNA Ends. Mol. Cel 8, 1129–1135. 10.1016/S1097-2765(01)00381-1 11741547

[B59] De Lorenzo S. B. Patel A. G. Hurley R. M. Kaufmann S. H. (2013). The Elephant and the Blind Men: Making Sense of PARP Inhibitors in Homologous Recombination Deficient Tumor Cells. Front. Oncol. 3. 10.3389/FONC.2013.00228 PMC3769628 24062981

[B60] Deans A. J. West S. C. (2011). DNA Interstrand Crosslink Repair and Cancer. Nat. Rev. Cancer 11, 467–480. 10.1038/NRC3088 21701511 PMC3560328

[B61] Dhar S. Gursoy-Yuzugullu O. Parasuram R. Price B. D. (2017). The Tale of a Tail: Histone H4 Acetylation and the Repair of DNA Breaks. Phil. Trans. R. Soc. B 372, 20160284. 10.1098/RSTB.2016.0284 28847821 PMC5577462

[B62] Dou H. Huang C. Van Nguyen T. Lu L.-S. Yeh E. T. H. (2011). SUMOylation and De-SUMOylation in Response to DNA Damage. FEBS Lett. 585, 2891–2896. 10.1016/j.febslet.2011.04.002 21486569

[B63] Drané P. Brault M.-E. Cui G. Meghani K. Chaubey S. Detappe A. (2017). TIRR Regulates 53BP1 by Masking its Histone Methyl-Lysine Binding Function. Nature 543, 211–216. 10.1038/NATURE21358 28241136 PMC5441565

[B64] Drenichev M. S. Mikhailov S. N. (2016). Poly(ADP-ribose): From Chemical Synthesis to Drug Design. Bioorg. Med. Chem. Lett. 26, 3395–3403. 10.1016/J.BMCL.2016.06.008 27318540

[B65] Dutta A. Eckelmann B. Adhikari S. Ahmed K. M. Sengupta S. Pandey A. (2017). Microhomology-mediated End Joining Is Activated in Irradiated Human Cells Due to Phosphorylation-dependent Formation of the XRCC1 Repair Complex. Nucleic Acids Res. 45, gkw1262. 10.1093/NAR/GKW1262 PMC5389627 27994036

[B66] Elbakry A. Löbrich M. (2021). Homologous Recombination Subpathways: A Tangle to Resolve. Front. Genet. 12, 1402. 10.3389/FGENE.2021.723847 PMC8365153 34408777

[B67] Everett R. D. (1987). The Regulation of Transcription of Viral and Cellular Genes by Herpesvirus Immediate-Early Gene Products (Review). Anticancer Res. 7, 589–604. Available at: https://pubmed.ncbi.nlm.nih.gov/3310848/ (Accessed September 10, 2021). 3310848

[B68] Falck J. Coates J. Jackson S. P. (2005). Conserved Modes of Recruitment of ATM, ATR and DNA-PKcs to Sites of DNA Damage. Nature 434, 605–611. 10.1038/nature03442 15758953

[B69] Feng L. Chen J. (2012). The E3 Ligase RNF8 Regulates KU80 Removal and NHEJ Repair. Nat. Struct. Mol. Biol. 19, 201–206. 10.1038/nsmb.2211 22266820 PMC3888515

[B70] Feng X. Koh D. W. (2013). Roles of Poly(ADP-Ribose) Glycohydrolase in DNA Damage and Apoptosis. Int. Rev. Cel Mol. Biol. 304, 227–281. 10.1016/B978-0-12-407696-9.00005-1 23809438

[B71] Fijen C. Rothenberg E. (2021). The Evolving Complexity of DNA Damage Foci: RNA, Condensates and Chromatin in DNA Double-Strand Break Repair. DNA Repair 105, 103170. 10.1016/J.DNAREP.2021.103170 34256335 PMC8364513

[B72] Findlay S. Heath J. Luo V. M. Malina A. Morin T. Coulombe Y. (2018). SHLD 2/FAM 35A Co‐operates with REV 7 to Coordinate DNA Double‐strand Break Repair Pathway Choice. EMBO J. 37. 10.15252/embj.2018100158 PMC6138439 30154076

[B73] Forment J. V. Kaidi A. Jackson S. P. (2012). Chromothripsis and Cancer: Causes and Consequences of Chromosome Shattering. Nat. Rev. Cancer 12, 663–670. 10.1038/nrc3352 22972457

[B74] Fouquerel E. Sobol R. W. (2014). ARTD1 (PARP1) Activation and NAD+ in DNA Repair and Cell Death. DNA Repair 23, 27–32. 10.1016/j.dnarep.2014.09.004 25283336 PMC4252787

[B75] Fradet-Turcotte A. Canny M. D. Escribano-Díaz C. Orthwein A. Leung C. C. Y. Huang H. (2013). 53BP1 Is a Reader of the DNA-Damage-Induced H2A Lys 15 Ubiquitin Mark. Nature 499, 50–54. 10.1038/NATURE12318 23760478 PMC3955401

[B76] Francia S. Michelini F. Saxena A. Tang D. de Hoon M. Anelli V. (2012). Site-specific DICER and DROSHA RNA Products Control the DNA-Damage Response. Nature 488, 231–235. 10.1038/nature11179 22722852 PMC3442236

[B77] Friedberg E. C. (2001). How Nucleotide Excision Repair Protects against Cancer. Nat. Rev. Cancer 1 (1), 22–33. 10.1038/35094000 11900249

[B78] Frit P. Ropars V. Modesti M. Charbonnier J. B. Calsou P. (2019). Plugged into the Ku-DNA Hub: The NHEJ Network. Prog. Biophys. Mol. Biol. 147, 62–76. 10.1016/j.pbiomolbio.2019.03.001 30851288

[B79] Galande S. Kohwi-Shigematsu T. (1999). Poly(ADP-ribose) Polymerase and Ku Autoantigen Form a Complex and Synergistically Bind to Matrix Attachment Sequences. J. Biol. Chem. 274, 20521–20528. 10.1074/JBC.274.29.20521 10400681

[B80] Ghezraoui H. Oliveira C. Becker J. R. Bilham K. Moralli D. Anzilotti C. (2018). 53BP1 Cooperation with the REV7-Shieldin Complex Underpins DNA Structure-specific NHEJ. Nature 560, 122–127. 10.1038/S41586-018-0362-1 30046110 PMC6989217

[B81] Goldberg M. Stucki M. Falck J. D'Amours D. Rahman D. Pappin D. (2003). MDC1 Is Required for the Intra-S-phase DNA Damage Checkpoint. Nature 421, 952–956. 10.1038/nature01445 12607003

[B82] Goodarzi A. A. Kurka T. Jeggo P. A. (2011). KAP-1 Phosphorylation Regulates CHD3 Nucleosome Remodeling during the DNA Double-Strand Break Response. Nat. Struct. Mol. Biol. 18, 831–839. 10.1038/NSMB.2077 21642969

[B83] Guiducci G. Stojic L. (2021). Long Noncoding RNAs at the Crossroads of Cell Cycle and Genome Integrity. Trends Genet. 37, 528–546. 10.1016/J.TIG.2021.01.006 33685661

[B84] Guo X. Bai Y. Zhao M. Zhou M. Shen Q. Yun C.-H. (2018). Acetylation of 53BP1 Dictates the DNA Double Strand Break Repair Pathway. Nucleic Acids Res. 46, 689–703. 10.1093/nar/gkx1208 29190394 PMC5778472

[B85] Gupta R. Somyajit K. Narita T. Maskey E. Stanlie A. Kremer M. (2018). DNA Repair Network Analysis Reveals Shieldin as a Key Regulator of NHEJ and PARP Inhibitor Sensitivity. Cell 173, 972–988. 10.1016/j.cell.2018.03.050 29656893 PMC8108093

[B86] Ha G.-H. Ji J.-H. Chae S. Park J. Kim S. Lee J.-K. (2019). Pellino1 Regulates Reversible ATM Activation via NBS1 Ubiquitination at DNA Double-Strand Breaks. Nat. Commun. 10. 10.1038/s41467-019-09641-9 PMC6450972 30952868

[B87] Haince J.-F. McDonald D. Rodrigue A. Déry U. Masson J.-Y. Hendzel M. J. (2008). PARP1-dependent Kinetics of Recruitment of MRE11 and NBS1 Proteins to Multiple DNA Damage Sites. J. Biol. Chem. 283, 1197–1208. 10.1074/jbc.M706734200 18025084

[B88] Hanscom T. McVey M. (2020). Regulation of Error-Prone DNA Double-Strand Break Repair and its Impact on Genome Evolution. Cells 9, 1657. 10.3390/CELLS9071657 PMC7407515 32660124

[B89] Hanzlikova H. Gittens W. Krejcikova K. Zeng Z. Caldecott K. W. (2017). Overlapping Roles for PARP1 and PARP2 in the Recruitment of Endogenous XRCC1 and PNKP into Oxidized Chromatin. Nucleic Acids Res. 45, gkw1246–2557. 10.1093/NAR/GKW1246 PMC5389470 27965414

[B90] Hartlerode A. J. Morgan M. J. Wu Y. Buis J. Ferguson D. O. (2015). Recruitment and Activation of the ATM Kinase in the Absence of DNA-Damage Sensors. Nat. Struct. Mol. Biol. 22, 736–743. 10.1038/nsmb.3072 26280532 PMC4560612

[B91] Hausmann M. Wagner E. Lee J.-H. Schrock G. Schaufler W. Krufczik M. (2018). Super-resolution Localization Microscopy of Radiation-Induced Histone H2AX-Phosphorylation in Relation to H3K9-Trimethylation in HeLa Cells. Nanoscale 10, 4320–4331. 10.1039/c7nr08145f 29443341

[B92] He Y. J. Meghani K. Caron M.-C. Yang C. Ronato D. A. Bian J. (2018). DYNLL1 Binds to MRE11 to Limit DNA End Resection in BRCA1-Deficient Cells. Nature 563, 522–526. 10.1038/S41586-018-0670-5 30464262 PMC7155769

[B93] Helfricht A. Wiegant W. Thijssen P. Vertegaal A. Luijsterburg M. van Attikum H. (2013). Remodeling and Spacing Factor 1 (RSF1) Deposits Centromere Proteins at DNA Double-Strand Breaks to Promote Non-homologous End-Joining. Cell Cycle 12, 3070–3082. 10.4161/CC.26033 23974106 PMC3875681

[B94] Helleday T. (2011). The Underlying Mechanism for the PARP and BRCA Synthetic Lethality: Clearing up the Misunderstandings. Mol. Oncol. 5, 387–393. 10.1016/J.MOLONC.2011.07.001 21821475 PMC5528309

[B95] Henriksson S. Rassoolzadeh H. Hedström E. Coucoravas C. Julner A. Goldstein M. (2014). The Scaffold Protein WRAP53β Orchestrates the Ubiquitin Response Critical for DNA Double-Strand Break Repair. Genes Dev. 28, 2726–2738. 10.1101/gad.246546.114 25512560 PMC4265676

[B96] Her J. Soo Lee N. Kim Y. Kim H. (2016). Factors Forming the BRCA1-A Complex Orchestrate BRCA1 Recruitment to the Sites of DNA Damage. Acta Biochim. Biophys. Sin 48, 658–664. 10.1093/ABBS/GMW047 27325824

[B97] Hoeijmakers J. H. J. (2009). DNA Damage, Aging, and Cancer. N. Engl. J. Med. 361, 1475–1485. 10.1056/NEJMra0804615 19812404

[B98] Horton J. K. Stefanick D. F. Prasad R. Gassman N. R. Kedar P. S. Wilson S. H. (2014). Base Excision Repair Defects Invoke Hypersensitivity to PARP Inhibition. Mol. Cancer Res. 12, 1128–1139. 10.1158/1541-7786.MCR-13-0502 24770870 PMC4135006

[B99] Hu Q. Botuyan M. V. Zhao D. Cui G. Mer E. Mer G. (2021). Mechanisms of BRCA1-BARD1 Nucleosome Recognition and Ubiquitylation. Nature 596, 438–443. 10.1038/s41586-021-03716-8 34321665 PMC8680157

[B100] Hu Y. Scully R. Sobhian B. Xie A. Shestakova E. Livingston D. M. (2011). RAP80-directed Tuning of BRCA1 Homologous Recombination Function at Ionizing Radiation-Induced Nuclear Foci. Genes Dev. 25, 685–700. 10.1101/GAD.2011011 21406551 PMC3070932

[B101] Ikura M. Furuya K. Fukuto A. Matsuda R. Adachi J. Matsuda T. (2016). Coordinated Regulation of TIP60 and Poly(ADP-Ribose) Polymerase 1 in Damaged-Chromatin Dynamics. Mol. Cel. Biol. 36, 1595–1607. 10.1128/mcb.01085-15 PMC4859686 26976643

[B102] Iliakis G. Mladenov E. Mladenova V. (2019). Necessities in the Processing of DNA Double Strand Breaks and Their Effects on Genomic Instability and Cancer. Cancers 11, 1671. 10.3390/CANCERS11111671 PMC6896103 31661831

[B103] Iliakis G. Murmann T. Soni A. (2015). Alternative End-Joining Repair Pathways Are the Ultimate Backup for Abrogated Classical Non-homologous End-Joining and Homologous Recombination Repair: Implications for the Formation of Chromosome Translocations. Mutat. Research/Genetic Toxicol. Environ. Mutagenesis 793, 166–175. 10.1016/J.MRGENTOX.2015.07.001 26520387

[B104] Ingram S. P. Warmenhoven J. W. Henthorn N. T. Smith E. A. K. Chadwick A. L. Burnet N. G. (2019). Mechanistic Modelling Supports Entwined rather Than Exclusively Competitive DNA Double-Strand Break Repair Pathway. Sci. Rep. 9, 1–13. 10.1038/s41598-019-42901-8 31015540 PMC6478946

[B105] Isabelle M. Moreel X. Gagné J.-P. Rouleau M. Ethier C. Gagné P. (2010). Investigation of PARP-1, PARP-2, and PARG Interactomes by Affinity-Purification Mass Spectrometry. Proteome Sci. 8, 22. 10.1186/1477-5956-8-22 20388209 PMC2861645

[B106] Ismail I. H. Gagné J.-P. Genois M.-M. Strickfaden H. Mcdonald D. Xu Z. (2015). The RNF138 E3 Ligase Displaces Ku to Promote DNA End Resection and Regulate DNA Repair Pathway Choice. Nat. Cel Biol. 17, 1446–1457. 10.1038/ncb3259 26502055

[B107] Jackson S. P. Bartek J. (2009). The DNA-Damage Response in Human Biology and Disease. Nature 461, 1071–1078. 10.1038/nature08467 19847258 PMC2906700

[B108] Jackson S. P. (2002). Sensing and Repairing DNA Double-Strand Breaks. Carcinogenesis 23, 687–696. 10.1093/carcin/23.5.687 12016139

[B109] Jacquet K. Fradet-Turcotte A. Avvakumov N. Lambert J.-P. Roques C. Pandita R. K. (2016). The TIP60 Complex Regulates Bivalent Chromatin Recognition by 53BP1 through Direct H4K20me Binding and H2AK15 Acetylation. Mol. Cel 62, 409–421. 10.1016/J.MOLCEL.2016.03.031 PMC4887106 27153538

[B110] Jakob B. Splinter J. Conrad S. Voss K.-O. Zink D. Durante M. (2011). DNA Double-Strand Breaks in Heterochromatin Elicit Fast Repair Protein Recruitment, Histone H2AX Phosphorylation and Relocation to Euchromatin. Nucleic Acids Res. 39, 6489–6499. 10.1093/nar/gkr230 21511815 PMC3159438

[B111] Jannetti S. A. Zeglis B. M. Zalutsky M. R. Reiner T. (2020). Poly(ADP-Ribose)Polymerase (PARP) Inhibitors and Radiation Therapy. Front. Pharmacol. 11, 170. 10.3389/FPHAR.2020.00170/BIBTEX 32194409 PMC7062869

[B112] Jeggo P. A. Löbrich M. (2007). DNA Double-Strand Breaks: Their Cellular and Clinical Impact? Oncogene 26, 7717–7719. 10.1038/sj.onc.1210868 18066083

[B113] Jeggo P. A. Löbrich M. (2017). DNA Non-homologous End-Joining Enters the Resection arena. Oncotarget 8, 93317–93318. 10.18632/ONCOTARGET.22075 29212151 PMC5706797

[B114] Jimeno S. Balestra F. R. Huertas P. (2021). The Emerging Role of RNA Modifications in DNA Double-Strand Break Repair. Front. Mol. Biosci. 8, 309. 10.3389/FMOLB.2021.664872 PMC8116738 33996910

[B115] Jiricny J. (2006). The Multifaceted Mismatch-Repair System. Nat. Rev. Mol. Cel Biol. 7 (7), 335–346. 10.1038/nrm1907 16612326

[B116] Jorgensen S. Schotta G. Sorensen C. S. (2013). Histone H4 Lysine 20 Methylation: Key Player in Epigenetic Regulation of Genomic Integrity. Nucleic Acids Res. 41, 2797–2806. 10.1093/NAR/GKT012 23345616 PMC3597678

[B117] Jungmichel S. Clapperton J. A. Lloyd J. Hari F. J. Spycher C. Pavic L. (2012). The Molecular Basis of ATM-dependent Dimerization of the Mdc1 DNA Damage Checkpoint Mediator. Nucleic Acids Res. 40, 3913–3928. 10.1093/nar/gkr1300 22234878 PMC3351161

[B118] Kelich J. M. Papaioannou H. Skordalakes E. (2021). Pol α-primase Dependent Nuclear Localization of the Mammalian CST Complex. Commun. Biol. 4 (4), 1. 10.1038/s42003-021-01845-4 33731801 PMC7969954

[B119] Ketley R. F. Gullerova M. (2020). Jack of All Trades? the Versatility of RNA in DNA Double-Strand Break Repair. Essays Biochem. 64, 721–735. 10.1042/EBC20200008 32618336 PMC7592198

[B120] Khanna K. K. Jackson S. P. (2001). DNA Double-Strand Breaks: Signaling, Repair and the Cancer Connection. Nat. Genet. 27, 247–254. 10.1038/85798 11242102

[B121] Khanna K. Lavin M. Jackson S. Mulhern T. (2001). ATM, a central Controller of Cellular Responses to DNA Damage. Cell Death Differ 8, 1052–1065. 10.1038/sj.cdd.4400874 11687884

[B122] Khurana S. Kruhlak M. J. Kim J. Tran A. D. Liu J. Nyswaner K. (2014). A Macrohistone Variant Links Dynamic Chromatin Compaction to BRCA1-dependent Genome Maintenance. Cel Rep. 8, 1049–1062. 10.1016/J.CELREP.2014.07.024 PMC4154351 25131201

[B123] Kilic S. Lezaja A. Gatti M. Bianco E. Michelena J. Imhof R. (2019). Phase Separation of 53 BP 1 Determines Liquid‐like Behavior of DNA Repair Compartments. EMBO J. 38. 10.15252/EMBJ.2018101379 PMC6694294 31267591

[B124] Kozlov S. V. Graham M. E. Jakob B. Tobias F. Kijas A. W. Tanuji M. (2011). Autophosphorylation and ATM Activation. J. Biol. Chem. 286, 9107–9119. 10.1074/JBC.M110.204065 21149446 PMC3059052

[B125] Kozlov S. V. Graham M. E. Peng C. Chen P. Robinson P. J. Lavin M. F. (2006). Involvement of Novel Autophosphorylation Sites in ATM Activation. EMBO J. 25, 3504–3514. 10.1038/SJ.EMBOJ.7601231 16858402 PMC1538573

[B126] Kramara J. Osia B. Malkova A. (2018). Break-Induced Replication: The where, the Why, and the How. Trends Genet. 34, 518–531. 10.1016/j.tig.2018.04.002 29735283 PMC6469874

[B127] Krenning L. van den Berg J. Medema R. H. (2019). Life or Death after a Break: What Determines the Choice? Mol. Cel 76, 346–358. 10.1016/j.molcel.2019.08.023 31561953

[B128] Krokan H. E. Bjoras M. (2013). Base Excision Repair. Cold Spring Harbor Perspect. Biol. 5, a012583. 10.1101/cshperspect.a012583 PMC3683898 23545420

[B129] Krüger A. Bürkle A. Hauser K. Mangerich A. (2020). Real-time Monitoring of PARP1-dependent PARylation by ATR-FTIR Spectroscopy. Nat. Commun. 11, 1–15. 10.1038/s41467-020-15858-w 32358582 PMC7195430

[B130] Kumar A. Kono H. (2020). Heterochromatin Protein 1 (HP1): Interactions with Itself and Chromatin Components. Biophys. Rev. 12, 387–400. 10.1007/s12551-020-00663-y 32144738 PMC7242596

[B131] Langelier M.-F. Billur R. Sverzhinsky A. Black B. E. Pascal J. M. (2021). HPF1 Dynamically Controls the PARP1/2 Balance between Initiating and Elongating ADP-Ribose Modifications. Nat. Commun. 12, 1. 10.1038/s41467-021-27043-8 34795260 PMC8602370

[B132] Langelier M.-F. Pascal J. M. (2013). PARP-1 Mechanism for Coupling DNA Damage Detection to poly(ADP-Ribose) Synthesis. Curr. Opin. Struct. Biol. 23, 134–143. 10.1016/J.SBI.2013.01.003 23333033 PMC3572337

[B133] Langelier M.-F. Planck J. L. Roy S. Pascal J. M. (2012). Structural Basis for DNA Damage-dependent poly(ADP-Ribosyl)ation by Human PARP-1. Science 336, 728–732. 10.1126/science.1216338 22582261 PMC3532513

[B134] Langerak P. Mejia-Ramirez E. Limbo O. Russell P. (2011). Release of Ku and MRN from DNA Ends by Mre11 Nuclease Activity and Ctp1 Is Required for Homologous Recombination Repair of Double-Strand Breaks. Plos Genet. 7, e1002271. 10.1371/JOURNAL.PGEN.1002271 21931565 PMC3169521

[B135] Lans H. Marteijn J. A. Vermeulen W. (2012). ATP-dependent Chromatin Remodeling in the DNA-Damage Response. Epigenetics & Chromatin 5, 1–14. 10.1186/1756-8935-5-4 22289628 PMC3275488

[B136] Lavin M. Kozlov S. Gatei M. Kijas A. (2015). ATM-dependent Phosphorylation of All Three Members of the MRN Complex: From Sensor to Adaptor. Biomolecules 5, 2877–2902. 10.3390/biom5042877 26512707 PMC4693261

[B137] Lee K. K. Workman J. L. (2007). Histone Acetyltransferase Complexes: One Size Doesn't Fit All. Nat. Rev. Mol. Cel Biol. 8 (8), 284–295. 10.1038/nrm2145 17380162

[B138] Leung A. K. L. (2020). Poly(ADP-ribose): A Dynamic Trigger for Biomolecular Condensate Formation. Trends Cel Biol. 30, 370–383. 10.1016/j.tcb.2020.02.002 PMC7326565 32302549

[B139] Leung C. C. Y. Glover J. N. M. (2011). BRCT Domains. Cell Cycle 10, 2461–2470. 10.4161/cc.10.15.16312 21734457 PMC3180187

[B140] Li M. Lu L.-Y. Yang C.-Y. Wang S. Yu X. (2013). The FHA and BRCT Domains Recognize ADP-Ribosylation during DNA Damage Response. Genes Dev. 27, 1752–1768. 10.1101/gad.226357.113 23964092 PMC3759693

[B141] Li M. Yu X. (2013). Function of BRCA1 in the DNA Damage Response Is Mediated by ADP-Ribosylation. Cancer Cell 23, 693–704. 10.1016/J.CCR.2013.03.025 23680151 PMC3759356

[B142] Li X. Heyer W.-D. (2008). Homologous Recombination in DNA Repair and DNA Damage Tolerance. Cell Res 18, 99–113. 10.1038/cr.2008.1 18166982 PMC3087377

[B143] Lim C. J. Barbour A. T. Zaug A. J. Goodrich K. J. McKay A. E. Wuttke D. S. (2020). The Structure of Human CST Reveals a Decameric Assembly Bound to Telomeric DNA. Science 368, 1081–1085. 10.1126/SCIENCE.AAZ9649 32499435 PMC7559292

[B144] Lindahl T. Barnes D. E. (2000). Repair of Endogenous DNA Damage. Cold Spring Harbor Symposia Quantitative Biol. 65, 127–134. 10.1101/SQB.2000.65.127 12760027

[B145] Lindahl T. (1993). Instability and Decay of the Primary Structure of DNA. Nature 362, 709–715. 10.1038/362709a0 8469282

[B146] Liu C. Vyas A. Kassab M. A. Singh A. K. Yu X. (2017). The Role of Poly ADP-Ribosylation in the First Wave of DNA Damage Response. Nucleic Acids Res. 45, 8129–8141. 10.1093/nar/gkx565 28854736 PMC5737498

[B147] Liu J. Luo S. Zhao H. Liao J. Li J. Yang C. (2012). Structural Mechanism of the Phosphorylation-dependent Dimerization of the MDC1 Forkhead-Associated Domain. Nucleic Acids Res. 40, 3898–3912. 10.1093/nar/gkr1296 22234877 PMC3351156

[B148] Liu S. Hua Y. Wang J. Li L. Yuan J. Zhang B. (2021). RNA Polymerase III Is Required for the Repair of DNA Double-Strand Breaks by Homologous Recombination. Cell 184, 1314–1329. 10.1016/J.CELL.2021.01.048 33626331

[B149] Llorens-Agost M. Ensminger M. Le H. P. Gawai A. Liu J. Cruz-García A. (2021). Polθ-Mediated End Joining Is Restricted by RAD52 and BRCA2 until the Onset of Mitosis. Nat. Cel Biol. 23, 1095–1104. 10.1038/s41556-021-00764-0 PMC8675436 34616022

[B150] Löbrich M. Jeggo P. (2017). A Process of Resection-dependent Nonhomologous End Joining Involving the Goddess Artemis. Trends Biochem. Sci. 42, 690–701. 10.1016/j.tibs.2017.06.011 28739276 PMC5604544

[B151] Lok G. T.-M. Sy S. M.-H. Dong S.-S. Ching Y.-P. Tsao S. W. Thomson T. M. (2012). Differential Regulation of RNF8-Mediated Lys48- and Lys63-Based Poly-Ubiquitylation. Nucleic Acids Res. 40, 196–205. 10.1093/nar/gkr655 21911360 PMC3245915

[B152] Lu C.-S. Truong L. N. Aslanian A. Shi L. Z. Li Y. Hwang P. Y.-H. (2012). The RING finger Protein RNF8 Ubiquitinates Nbs1 to Promote DNA Double-Strand Break Repair by Homologous Recombination. J. Biol. Chem. 287, 43984–43994. 10.1074/jbc.M112.421545 23115235 PMC3527981

[B153] Luessing J. Sakhteh M. Sarai N. Frizzell L. Tsanov N. Ramberg K. O. (2021). The Nuclear Kinesin KIF18B Promotes 53BP1-Mediated DNA Double-Strand Break Repair. Cel Rep. 35, 109306. 10.1016/j.celrep.2021.109306 34192545

[B154] Luijsterburg M. S. de Krijger I. Wiegant W. W. Shah R. G. Smeenk G. de Groot A. J. L. (2016). PARP1 Links CHD2-Mediated Chromatin Expansion and H3.3 Deposition to DNA Repair by Non-homologous End-Joining. Mol. Cel 61, 547–562. 10.1016/j.molcel.2016.01.019 PMC4769320 26895424

[B155] Luijsterburg M. S. Dinant C. Lans H. Stap J. Wiernasz E. Lagerwerf S. (2009). Heterochromatin Protein 1 Is Recruited to Various Types of DNA Damage. J. Cel Biol. 185, 577–586. 10.1083/JCB.200810035 PMC2711568 19451271

[B156] Luo K. Yuan J. Lou Z. (2011). Oligomerization of MDC1 Protein Is Important for Proper DNA Damage Response. J. Biol. Chem. 286, 28192–28199. 10.1074/jbc.M111.258087 21705321 PMC3151064

[B157] Mallette F. A. Mattiroli F. Cui G. Young L. C. Hendzel M. J. Mer G. (2012). RNF8- and RNF168-dependent Degradation of KDM4A/JMJD2A Triggers 53BP1 Recruitment to DNA Damage Sites. EMBO J. 31, 1865–1878. 10.1038/emboj.2012.47 22373579 PMC3343333

[B158] Maltseva E. A. Krasikova Y. S. Sukhanova M. V. Rechkunova N. I. Lavrik O. I. (2018). Replication Protein A as a Modulator of the poly(ADP-Ribose)polymerase 1 Activity. DNA Repair 72, 28–38. 10.1016/J.DNAREP.2018.09.010 30291044

[B159] Mansour W. Y. Rhein T. Dahm-Daphi J. (2010). The Alternative End-Joining Pathway for Repair of DNA Double-Strand Breaks Requires PARP1 but Is Not Dependent upon Microhomologies. Nucleic Acids Res. 38, 6065–6077. 10.1093/NAR/GKQ387 20483915 PMC2952854

[B160] Marchal C. Sima J. Gilbert D. M. (2019). Control of DNA Replication Timing in the 3D Genome. Nat. Rev. Mol. Cel Biol. 20, 721–737. 10.1038/s41580-019-0162-y PMC11567694 31477886

[B161] Maréchal A. Zou L. (2013). DNA Damage Sensing by the ATM and ATR Kinases. Cold Spring Harb. Perspect. Biol. 5. 10.1586/14737175.4.6.935 PMC3753707 24003211

[B162] Mari P.-O. Florea B. I. Persengiev S. P. Verkaik N. S. Bruggenwirth H. T. Modesti M. (2006). Dynamic Assembly of End-Joining Complexes Requires Interaction between Ku70/80 and XRCC4. Proc. Natl. Acad. Sci. 103, 18597–18602. 10.1073/PNAS.0609061103 17124166 PMC1693708

[B163] Marnef A. Legube G. (2021). R-loops as Janus-Faced Modulators of DNA Repair. Nat. Cel Biol. 23, 305–313. 10.1038/s41556-021-00663-4 33837288

[B164] Masson M. Niedergang C. Schreiber V. Muller S. Menissier-de Murcia J. de Murcia G. (1998). XRCC1 Is Specifically Associated with poly(ADP-Ribose) Polymerase and Negatively Regulates its Activity Following DNA Damage. Mol. Cel. Biol. 18, 3563–3571. 10.1128/MCB.18.6.3563 PMC108937 9584196

[B270] Matsuoka S. Ballif B. A. Smogorzewska A. McDonald E. R. Hurov K. E. Luo J. (2007). ATM And ATR Substrate Analysis Reveals Extensive Protein Networks Responsive To DNA Damage. Sci. 316, 1160–1166. 10.1126/SCIENCE.1140321 17525332

[B165] Mattiroli F. Vissers J. H. A. van Dijk W. J. Ikpa P. Citterio E. Vermeulen W. (2012). RNF168 Ubiquitinates K13-15 on H2A/H2AX to Drive DNA Damage Signaling. Cell 150, 1182–1195. 10.1016/j.cell.2012.08.005 22980979

[B166] Mazina O. M. Keskin H. Hanamshet K. Storici F. Mazin A. V. (2017). Rad52 Inverse Strand Exchange Drives RNA-Templated DNA Double-Strand Break Repair. Mol. Cel 67, 19–29. 10.1016/J.MOLCEL.2017.05.019 PMC5547995 28602639

[B167] Meers C. Keskin H. Storici F. (2016). DNA Repair by RNA: Templated, or Not Templated, that Is the Question. DNA Repair 44, 17–21. 10.1016/J.DNAREP.2016.05.002 27237587 PMC4958532

[B168] Mehta A. Haber J. E. (2014). Sources of DNA Double-Strand Breaks and Models of Recombinational DNA Repair. Cold Spring Harbor Perspect. Biol. 6, a016428. 10.1101/cshperspect.a016428 PMC4142968 25104768

[B169] Melander F. Bekker-Jensen S. Falck J. Bartek J. Mailand N. Lukas J. (2008). Phosphorylation of SDT Repeats in the MDC1 N Terminus Triggers Retention of NBS1 at the DNA Damage-Modified Chromatin. J. Cel Biol. 181, 213–226. 10.1083/jcb.200708210 PMC2315670 18411307

[B170] Min S. Jo S. Lee H.-S. Chae S. Lee J.-S. Ji J.-H. (2014). ATM-dependent Chromatin Remodeler Rsf-1 Facilitates DNA Damage Checkpoints and Homologous Recombination Repair. Cell Cycle 13, 666–677. 10.4161/CC.27548 24351651

[B171] Mirman Z. de Lange T. (2020). 53BP1: a DSB Escort. Genes Dev. 34, 7–23. 10.1101/gad.333237.119 31896689 PMC6938671

[B172] Mirman Z. Lottersberger F. Takai H. Kibe T. Gong Y. Takai K. (2018). 53BP1-RIF1-shieldin Counteracts DSB Resection through CST- and Polα-dependent Fill-In. Nature 560, 112–116. 10.1038/s41586-018-0324-7 30022158 PMC6072559

[B173] Monaghan L. Massett M. E. Bunschoten R. P. Hoose A. Pirvan P.-A. Liskamp R. M. J. (2019). The Emerging Role of H3K9me3 as a Potential Therapeutic Target in Acute Myeloid Leukemia. Front. Oncol. 9. 10.3389/fonc.2019.00705 PMC6687838 31428579

[B174] Morris J. R. (2021). Is it a Wrap? Nucleosome Interactions of the BRCA1-Binding Partner, BARD1, Steal the Scene. Nat. Struct. Mol. Biol. 28, 708–710. 10.1038/s41594-021-00658-7 34518696

[B175] Moyal L. Lerenthal Y. Gana-Weisz M. Mass G. So S. Wang S.-Y. (2011). Requirement of ATM-dependent Monoubiquitylation of Histone H2B for Timely Repair of DNA Double-Strand Breaks. Mol. Cel 41, 529–542. 10.1016/j.molcel.2011.02.015 PMC3397146 21362549

[B269] Mu J. J. Wang Y. Luo H. Leng M. Zhang J. Yang T. (2007). A Proteomic Analysis Of Ataxia Telangiectasia-Mutated (ATM)/ATM-Rad3-Related (ATR) Substrates Identifies The Ubiquitin-Proteasome System As A Regulator For DNA Damage Checkpoints. J. Biol. Chem. 282, 17330–17334. 10.1074/JBC.C700079200 17478428

[B176] Murai J. Huang S.-y. N. Das B. B. Renaud A. Zhang Y. Doroshow J. H. (2012). Trapping of PARP1 and PARP2 by Clinical PARP Inhibitors. Cancer Res. 72, 5588–5599. 10.1158/0008-5472.CAN-12-2753 23118055 PMC3528345

[B177] Murata M. M. Kong X. Moncada E. Chen Y. Imamura H. Wang P. (2019). NAD+ Consumption by PARP1 in Response to DNA Damage Triggers Metabolic Shift Critical for Damaged Cell Survival. MBoC 30, 2584–2597. 10.1091/MBC.E18-10-0650 31390283 PMC6740200

[B178] Myler L. R. Gallardo I. F. Soniat M. M. Deshpande R. A. Gonzalez X. B. Kim Y. (2017). Single-Molecule Imaging Reveals How Mre11-Rad50-Nbs1 Initiates DNA Break Repair. Mol. Cel 67, 891–898. 10.1016/j.molcel.2017.08.002 PMC5609712 28867292

[B179] Nakamura K. Saredi G. Becker J. R. Foster B. M. Nguyen N. V. Beyer T. E. (2019). H4K20me0 Recognition by BRCA1-BARD1 Directs Homologous Recombination to Sister Chromatids. Nat. Cel Biol. 21, 311–318. 10.1038/s41556-019-0282-9 PMC6420097 30804502

[B180] Nowsheen S. Aziz K. Aziz A. Deng M. Qin B. Luo K. (2018). L3MBTL2 Orchestrates Ubiquitin Signalling by Dictating the Sequential Recruitment of RNF8 and RNF168 after DNA Damage. Nat. Cel Biol. 20, 455–464. 10.1038/s41556-018-0071-x PMC6083879 29581593

[B181] Ochs F. Karemore G. Miron E. Brown J. Sedlackova H. Rask M.-B. (2019). Stabilization of Chromatin Topology Safeguards Genome Integrity. Nature 574, 571–574. 10.1038/s41586-019-1659-4 31645724

[B182] Ochs F. Somyajit K. Altmeyer M. Rask M.-B. Lukas J. Lukas C. (2016). 53BP1 Fosters Fidelity of Homology-Directed DNA Repair. Nat. Struct. Mol. Biol. 23, 714–721. 10.1038/nsmb.3251 27348077

[B183] Onaka A. T. Su J. Katahira Y. Tang C. Zafar F. Aoki K. (2020). DNA Replication Machinery Prevents Rad52-dependent Single-Strand Annealing that Leads to Gross Chromosomal Rearrangements at Centromeres. Commun. Biol. 3. 10.1038/s42003-020-0934-0 PMC7193609 32355220

[B184] Onn L. Portillo M. Ilic S. Cleitman G. Stein D. Kaluski S. (2020). SIRT6 Is a DNA Double-Strand Break Sensor. Elife 9. 10.7554/eLife.51636 PMC7051178 31995034

[B185] Palancade B. Rothstein R. (2021). The Ultimate (Mis)match: When DNA Meets RNA. Cells 10, 1433. 10.3390/CELLS10061433 34201169 PMC8227541

[B186] Panday A. Willis N. A. Elango R. Menghi F. Duffey E. E. Liu E. T. (2021). FANCM Regulates Repair Pathway Choice at Stalled Replication forks. Mol. Cel 81, 2428–2444. e6. 10.1016/J.MOLCEL.2021.03.044 PMC8180084 33882298

[B187] Panier S. Boulton S. J. (2013). Double-strand Break Repair: 53BP1 Comes into Focus. Nat. Rev. Mol. Cel Biol. 15 (15), 7–18. 10.1038/nrm3719 24326623

[B188] Panier S. Ichijima Y. Fradet-Turcotte A. Leung C. C. Y. Kaustov L. Arrowsmith C. H. (2012). Tandem Protein Interaction Modules Organize the Ubiquitin-dependent Response to DNA Double-Strand Breaks. Mol. Cel 47, 383–395. 10.1016/J.MOLCEL.2012.05.045 22742833

[B189] Pascal J. M. (2018). The Comings and Goings of PARP-1 in Response to DNA Damage. DNA Repair 71, 177–182. 10.1016/J.DNAREP.2018.08.022 30177435 PMC6637744

[B190] Patel A. G. Sarkaria J. N. Kaufmann S. H. (2011). Nonhomologous End Joining Drives poly(ADP-Ribose) Polymerase (PARP) Inhibitor Lethality in Homologous Recombination-Deficient Cells. Proc. Natl. Acad. Sci. 108, 3406–3411. 10.1073/PNAS.1013715108/-/DCSUPPLEMENTAL 21300883 PMC3044391

[B191] Paull T. T. (2018). 20 Years of Mre11 Biology: No End in Sight. Mol. Cel 71, 419–427. 10.1016/J.MOLCEL.2018.06.033 30057197

[B192] Paull T. T. (2021). Reconsidering Pathway Choice: a Sequential Model of Mammalian DNA Double-Strand Break Pathway Decisions. Curr. Opin. Genet. Dev. 71, 55–62. 10.1016/J.GDE.2021.06.011 34293662 PMC8671155

[B193] Pei H. Zhang L. Luo K. Qin Y. Chesi M. Fei F. (2011). MMSET Regulates Histone H4K20 Methylation and 53BP1 Accumulation at DNA Damage Sites. Nature 470, 124–128. 10.1038/NATURE09658 21293379 PMC3064261

[B194] Pellegrino S. Michelena J. Teloni F. Imhof R. Altmeyer M. (2017). Replication-Coupled Dilution of H4K20me2 Guides 53BP1 to Pre-replicative Chromatin. Cel Rep. 19, 1819–1831. 10.1016/J.CELREP.2017.05.016 PMC5857200 28564601

[B195] Pessina F. Lowndes N. F. (2014). The RSF1 Histone-Remodelling Factor Facilitates DNA Double-Strand Break Repair by Recruiting Centromeric and Fanconi Anaemia Proteins. Plos Biol. 12, e1001856. 10.1371/JOURNAL.PBIO.1001856 24800743 PMC4011676

[B196] Pham N. Yan Z. Yu Y. Faria Afreen M. Malkova A. Haber J. E. (2021). Mechanisms Restraining Break‐induced Replication at Two‐ended DNA Double‐strand Breaks. EMBO J. 40, e104847. 10.15252/EMBJ.2020104847 33844333 PMC8126933

[B197] Poulsen M. Lukas C. Lukas J. Bekker-Jensen S. Mailand N. (2012). Human RNF169 Is a Negative Regulator of the Ubiquitin-dependent Response to DNA Double-Strand Breaks. J. Cel Biol. 197, 189–199. 10.1083/jcb.201109100 PMC3328375 22492721

[B198] Price B. D. D’Andrea A. D. (2013). Chromatin Remodeling at DNA Double-Strand Breaks. Cell 152, 1344–1354. 10.1016/j.cell.2013.02.011 23498941 PMC3670600

[B199] Qi Y. Warmenhoven J. W. Henthorn N. T. Ingram S. P. Xu X. G. Kirkby K. J. (2021). Mechanistic Modelling of Slow and Fast NHEJ DNA Repair Pathways Following Radiation for G0/G1 Normal Tissue Cells. Cancers 13, 2202. 10.3390/CANCERS13092202 34063683 PMC8124137

[B200] Qin B. Yu J. Nowsheen S. Wang M. Tu X. Liu T. (2019). UFL1 Promotes Histone H4 Ufmylation and ATM Activation. Nat. Commun. 10. 10.1038/s41467-019-09175-0 PMC6423285 30886146

[B201] Qin B. Yu J. Nowsheen S. Zhao F. Wang L. Lou Z. (2020). STK38 Promotes ATM Activation by Acting as a Reader of Histone H4 Ufmylation. Sci. Adv. 6. 10.1126/sciadv.aax8214 PMC7269669 32537488

[B202] Rajarajan P. Gil S. E. Brennand K. J. Akbarian S. (2016). Spatial Genome Organization and Cognition. Nat. Rev. Neurosci. 17, 681–691. 10.1038/nrn.2016.124 27708356 PMC5503467

[B203] Ramsden D. A. Carvajal-Garcia J. Gupta G. P. (2021). Mechanism, Cellular Functions and Cancer Roles of Polymerase-Theta-Mediated DNA End Joining. Nat. Rev. Mol. Cel Biol. 2021, 1–16. 10.1038/s41580-021-00405-2 PMC13537726 34522048

[B204] Rassoolzadeh H. Coucoravas C. Farnebo M. (2015). The Proximity Ligation Assay Reveals that at DNA Double-Strand Breaks WRAP53β Associates with γH2AX and Controls Interactions between RNF8 and MDC1. Nucleus 6, 417–424. 10.1080/19491034.2015.1106675 26734725 PMC4915514

[B205] Ray Chaudhuri A. Nussenzweig A. (2017). The Multifaceted Roles of PARP1 in DNA Repair and Chromatin Remodelling. Nat. Rev. Mol. Cel Biol. 18, 610–621. 10.1038/nrm.2017.53 PMC6591728 28676700

[B206] Riballo E. Kühne M. Rief N. Doherty A. Smith G. C. M. Recio M.-J. (2004). A Pathway of Double-Strand Break Rejoining Dependent upon ATM, Artemis, and Proteins Locating to γ-H2AX Foci. Mol. Cel 16, 715–724. 10.1016/J.MOLCEL.2004.10.029 15574327

[B207] Richard D. J. Bolderson E. Cubeddu L. Wadsworth R. I. M. Savage K. Sharma G. G. (2008). Single-stranded DNA-Binding Protein hSSB1 Is Critical for Genomic Stability. Nature 453, 677–681. 10.1038/nature06883 18449195

[B208] Richard D. J. Cubeddu L. Urquhart A. J. Bain A. Bolderson E. Menon D. (2011a). HSSB1 Interacts Directly with the MRN Complex Stimulating its Recruitment to DNA Double-Strand Breaks and its Endo-Nuclease Activity. Nucleic Acids Res. 39, 3643–3651. 10.1093/nar/gkq1340 21227926 PMC3089470

[B209] Richard D. J. Savage K. Bolderson E. Cubeddu L. So S. Ghita M. (2011b). HSSB1 Rapidly Binds at the Sites of DNA Double-Strand Breaks and Is Required for the Efficient Recruitment of the MRN Complex. Nucleic Acids Res. 39, 1692–1702. 10.1093/nar/gkq1098 21051358 PMC3061066

[B210] Ronato D. A. Mersaoui S. Y. Busatto F. F. Affar E. B. Richard S. Masson J.-Y. (2020). Limiting the DNA Double-Strand Break Resectosome for Genome Protection. Trends Biochem. Sci. 45, 779–793. 10.1016/j.tibs.2020.05.003 32513599

[B211] Rose M. Burgess J. T. O'Byrne K. Richard D. J. Bolderson E. (2020). PARP Inhibitors: Clinical Relevance, Mechanisms of Action and Tumor Resistance. Front. Cel Dev. Biol. 8, 564601. 10.3389/FCELL.2020.564601/BIBTEX PMC7509090 33015058

[B212] Rother M. B. Pellegrino S. Smith R. Gatti M. Meisenberg C. Wiegant W. W. (2020). CHD7 and 53BP1 Regulate Distinct Pathways for the Re-ligation of DNA Double-Strand Breaks. Nat. Commun. 11. 10.1038/s41467-020-19502-5 PMC7666215 33188175

[B213] Rulten S. L. Fisher A. E. O. Robert I. Zuma M. C. Rouleau M. Ju L. (2011). PARP-3 and APLF Function Together to Accelerate Nonhomologous End-Joining. Mol. Cel 41, 33–45. 10.1016/J.MOLCEL.2010.12.006 21211721

[B214] Rupnik A. Grenon M. Lowndes N. (2008). The MRN Complex. Curr. Biol. 18, R455–R457. 10.1016/j.cub.2008.03.040 18522810

[B215] Rupnik A. Lowndes N. F. Grenon M. (2009). MRN and the Race to the Break. Chromosoma 119, 115–135. 10.1007/S00412-009-0242-4 19862546

[B216] Salguero I. Belotserkovskaya R. Coates J. Sczaniecka-Clift M. Demir M. Jhujh S. (2019). MDC1 PST-Repeat Region Promotes Histone H2AX-independent Chromatin Association and DNA Damage Tolerance. Nat. Commun. 10. 10.1038/s41467-019-12929-5 PMC6858307 31729360

[B217] Saredi G. Huang H. Hammond C. M. Alabert C. Bekker-Jensen S. Forne I. (2016). H4K20me0 marks post-replicative Chromatin and Recruits the TONSL-Mms22l DNA Repair Complex. Nature 534, 714–718. 10.1038/nature18312 27338793 PMC4939875

[B218] Savitsky K. Bar-Shira A. Gilad S. Rotman G. Ziv Y. Vanagaite L. (1995). A Single Ataxia Telangiectasia Gene with a Product Similar to PI-3 Kinase. Science 268, 1749–1753. 10.1126/science.7792600 7792600

[B219] Scully R. Panday A. Elango R. Willis N. A. (2019). DNA Double-Strand Break Repair-Pathway Choice in Somatic Mammalian Cells. Nat. Rev. Mol. Cel Biol. 20, 698–714. 10.1038/s41580-019-0152-0 PMC7315405 31263220

[B220] Semlow D. R. Walter J. C. (2021). Mechanisms of Vertebrate DNA Interstrand Cross-Link Repair. Annu. Rev. Biochem. 90, 107–135. 10.1146/annurev-biochem-08032010.1146/annurev-biochem-080320-112510 33882259

[B221] Setiaputra D. Durocher D. (2019). Shieldin - the Protector of DNA Ends. EMBO Rep. 20. 10.15252/embr.201847560 PMC6501030 30948458

[B222] Shibata A. Jeggo P. (2019). A Historical Reflection on Our Understanding of Radiation-Induced DNA Double Strand Break Repair in Somatic Mammalian Cells; Interfacing the Past with the Present. Int. J. Radiat. Biol. 95, 945–956. 10.1080/09553002.2018.1564083 30608893

[B223] Shibata A. Jeggo P. A. (2020a). Roles for 53BP1 in the Repair of Radiation-Induced DNA Double Strand Breaks. DNA Repair 93, 102915. 10.1016/j.dnarep.2020.102915 33087281

[B224] Shibata A. Jeggo P. A. (2020b). Roles for the DNA-PK Complex and 53BP1 in Protecting Ends from Resection during DNA Double-Strand Break Repair. J. Radiat. Res. 61, 718–726. 10.1093/jrr/rraa053 32779701 PMC7482155

[B225] Shibata A. Jeggo P. Löbrich M. (2018). The Pendulum of the Ku-Ku Clock. DNA Repair 71, 164–171. 10.1016/j.dnarep.2018.08.020 30177438

[B226] Shiloh Y. (2003). ATM and Related Protein Kinases: Safeguarding Genome Integrity. Nat. Rev. Cancer 3, 155–168. 10.1038/nrc1011 12612651

[B227] Singh J. K. Smith R. Rother M. B. de Groot A. J. L. Wiegant W. W. Vreeken K. (2021). Zinc finger Protein ZNF384 Is an Adaptor of Ku to DNA during Classical Non-homologous End-Joining. Nat. Commun. 12 (12), 1–21. 10.1038/s41467-021-26691-0 34772923 PMC8589989

[B228] Spagnolo L. Barbeau J. Curtin N. J. Morris E. P. Pearl L. H. (2012). Visualization of a DNA-PK/PARP1 Complex. Nucleic Acids Res. 40, 4168–4177. 10.1093/NAR/GKR1231 22223246 PMC3351162

[B229] Spycher C. Miller E. S. Townsend K. Pavic L. Morrice N. A. Janscak P. (2008). Constitutive Phosphorylation of MDC1 Physically Links the MRE11-RAD50-NBS1 Complex to Damaged Chromatin. J. Cel Biol. 181, 227–240. 10.1083/JCB.200709008 PMC2315671 18411308

[B230] Stiff T. O’Driscoll M. Rief N. Iwabuchi K. Löbrich M. Jeggo P. A. (2004). ATM and DNA-PK Function Redundantly to Phosphorylate H2AX after Exposure to Ionizing Radiation. Cancer Res. 64, 2390–2396. 10.1158/0008-5472.CAN-03-3207 15059890

[B231] Storici F. Bebenek K. Kunkel T. A. Gordenin D. A. Resnick M. A. (2007). RNA-templated DNA Repair. Nature 447, 338–341. 10.1038/NATURE05720 17429354 PMC2121219

[B232] Strickfaden H. McDonald D. Kruhlak M. J. Haince J.-F. Th'ng J. P. H. Rouleau M. (2016). Poly(ADP-ribosyl)ation-dependent Transient Chromatin Decondensation and Histone Displacement Following Laser Microirradiation. J. Biol. Chem. 291, 1789–1802. 10.1074/jbc.M115.694992 26559976 PMC4722458

[B233] Sulkowski P. L. Oeck S. Dow J. Economos N. G. Mirfakhraie L. Liu Y. (2020). Oncometabolites Suppress DNA Repair by Disrupting Local Chromatin Signalling. Nature 582, 586–591. 10.1038/s41586-020-2363-0 32494005 PMC7319896

[B234] Sun Y. Jiang X. Chen S. Fernandes N. Price B. D. (2005). A Role for the Tip60 Histone Acetyltransferase in the Acetylation and Activation of ATM. Proc. Natl. Acad. Sci. 102, 13182–13187. 10.1073/PNAS.0504211102 16141325 PMC1197271

[B235] Sun Y. Jiang X. Price B. D. (2010). Tip60: Connecting Chromatin to DNA Damage Signaling. Cell Cycle 9, 930–936. 10.4161/CC.9.5.10931 20160506 PMC2901859

[B236] Sun Y. Jiang X. Xu Y. Ayrapetov M. K. Moreau L. A. Whetstine J. R. (2009). Histone H3 Methylation Links DNA Damage Detection to Activation of the Tumour Suppressor Tip60. Nat. Cel Biol. 11, 1376–1382. 10.1038/ncb1982 PMC2783526 19783983

[B237] Suskiewicz M. J. Zobel F. Ogden T. E. H. Fontana P. Ariza A. Yang J.-C. (2020). HPF1 Completes the PARP Active Site for DNA Damage-Induced ADP-Ribosylation. Nature 579, 598–602. 10.1038/s41586-020-2013-6 32028527 PMC7104379

[B238] Syed A. Tainer J. A. (2018). The MRE11-RAD50-NBS1 Complex Conducts the Orchestration of Damage Signaling and Outcomes to Stress in DNA Replication and Repair. Annu. Rev. Biochem. 87, 263–294. 10.1146/annurev-biochem-062917-012415 29709199 PMC6076887

[B239] Thorslund T. Ripplinger A. Hoffmann S. Wild T. Uckelmann M. Villumsen B. (2015). Histone H1 Couples Initiation and Amplification of Ubiquitin Signalling after DNA Damage. Nature 527, 389–393. 10.1038/nature15401 26503038

[B240] Tisi R. Vertemara J. Zampella G. Longhese M. P. (2020). Functional and Structural Insights into the MRX/MRN Complex, a Key Player in Recognition and Repair of DNA Double-Strand Breaks. Comput. Struct. Biotechnol. J. 18, 1137–1152. 10.1016/j.csbj.2020.05.013 32489527 PMC7260605

[B241] Tomasetti C. Vogelstein B. (2015). Variation in Cancer Risk Among Tissues Can Be Explained by the Number of Stem Cell Divisions. Science 347, 78–81. 10.1126/SCIENCE.1260825 25554788 PMC4446723

[B242] Uckelmann M. Sixma T. K. (2017). Histone Ubiquitination in the DNA Damage Response. DNA Repair 56, 92–101. 10.1016/j.dnarep.2017.06.011 28624371

[B243] van Beek L. McClay É. Patel S. Schimpl M. Spagnolo L. Maia de Oliveira T. (2021). PARP Power: A Structural Perspective on PARP1, PARP2, and PARP3 in DNA Damage Repair and Nucleosome Remodelling. Ijms 22, 5112. 10.3390/IJMS22105112 34066057 PMC8150716

[B244] van Sluis M. McStay B. (2017). Nucleolar Reorganization in Response to rDNA Damage. Curr. Opin. Cel Biol. 46, 81–86. 10.1016/J.CEB.2017.03.004 28431265

[B245] Verma P. Zhou Y. Cao Z. Deraska P. V. Deb M. Arai E. (2021). ALC1 Links Chromatin Accessibility to PARP Inhibitor Response in Homologous Recombination-Deficient Cells. Nat. Cel Biol. 23, 160–171. 10.1038/s41556-020-00624-3 PMC7880902 33462394

[B246] Walker J. R. Corpina R. A. Goldberg J. (2001). Structure of the Ku Heterodimer Bound to DNA and its Implications for Double-Strand Break Repair. Nature 412, 607–614. 10.1038/35088000 11493912

[B247] Walser F. Mulder M. P. C. Bragantini B. Burger S. Gubser T. Gatti M. (2020). Ubiquitin Phosphorylation at Thr12 Modulates the DNA Damage Response. Mol. Cel 80, 423–436. 10.1016/j.molcel.2020.09.017 PMC7655664 33022275

[B248] Wang H. Wang M. Wang H. Böcker W. Iliakis G. (2005). Complex H2AX Phosphorylation Patterns by Multiple Kinases Including ATM and DNA-PK in Human Cells Exposed to Ionizing Radiation and Treated with Kinase Inhibitors. J. Cel. Physiol. 202, 492–502. 10.1002/JCP.20141 15389585

[B249] Wang J. Aroumougame A. Lobrich M. Li Y. Chen D. Chen J. (2014). PTIP Associates with Artemis to Dictate DNA Repair Pathway Choice. Genes Dev. 28, 2693–2698. 10.1101/gad.252478.114 25512557 PMC4265673

[B250] Wang J. Yuan Z. Cui Y. Xie R. Yang G. Kassab M. A. (2018). Molecular Basis for the Inhibition of the Methyl-Lysine Binding Function of 53BP1 by TIRR. Nat. Commun. 9. 10.1038/S41467-018-05174-9 PMC6043480 30002377

[B251] Wang M. Wu W. Wu W. Rosidi B. Zhang L. Wang H. (2006). PARP-1 and Ku Compete for Repair of DNA Double Strand Breaks by Distinct NHEJ Pathways. Nucleic Acids Res. 34, 6170–6182. 10.1093/NAR/GKL840 17088286 PMC1693894

[B252] Wang Z. Gong Y. Peng B. Shi R. Fan D. Zhao H. (2019). MRE11 UFMylation Promotes ATM Activation. Nucleic Acids Res. 47, 4124–4135. 10.1093/nar/gkz110 30783677 PMC6486557

[B253] Wei H. Yu X. (2016). Functions of PARylation in DNA Damage Repair Pathways. Genomics, Proteomics & Bioinformatics 14, 131–139. 10.1016/j.gpb.2016.05.001 PMC4936651 27240471

[B254] Wei W. Ba Z. Gao M. Wu Y. Ma Y. Amiard S. (2012). A Role for Small RNAs in DNA Double-Strand Break Repair. Cell 149, 101–112. 10.1016/J.CELL.2012.03.002 22445173

[B255] Williams R. S. Dodson G. E. Limbo O. Yamada Y. Williams J. S. Guenther G. (2009). Nbs1 Flexibly Tethers Ctp1 and Mre11-Rad50 to Coordinate DNA Double-Strand Break Processing and Repair. Cell 139, 87–99. 10.1016/J.CELL.2009.07.033 19804755 PMC2762657

[B256] Woodbine L. Brunton H. Goodarzi A. A. Shibata A. Jeggo P. A. (2011). Endogenously Induced DNA Double Strand Breaks Arise in Heterochromatic DNA Regions and Require Ataxia Telangiectasia Mutated and Artemis for Their Repair. Nucleic Acids Res. 39, 6986–6997. 10.1093/NAR/GKR331 21596788 PMC3167608

[B257] Wright R. H. G. Lioutas A. Le Dily F. Soronellas D. Pohl A. Bonet J. (2016). ADP-ribose-derived Nuclear ATP Synthesis by NUDIX5 Is Required for Chromatin Remodeling. Science 352, 1221–1225. 10.1126/SCIENCE.AAD9335 27257257

[B258] Wu J. Zhang X. Zhang L. Wu C.-Y. Rezaeian A. H. Chan C.-H. (2012). Skp2 E3 Ligase Integrates ATM Activation and Homologous Recombination Repair by Ubiquitinating NBS1. Mol. Cel 46, 351–361. 10.1016/j.molcel.2012.02.018 PMC3518281 22464731

[B259] Wyatt D. W. Feng W. Conlin M. P. Yousefzadeh M. J. Roberts S. A. Mieczkowski P. (2016). Essential Roles for Polymerase θ-Mediated End Joining in the Repair of Chromosome Breaks. Mol. Cel 63, 662–673. 10.1016/J.MOLCEL.2016.06.020 PMC4992412 27453047

[B260] Yang G. Liu C. Chen S.-H. Kassab M. A. Hoff J. D. Walter N. G. (2018). Super-resolution Imaging Identifies PARP1 and the Ku Complex Acting as DNA Double-Strand Break Sensors. Nucleic Acids Res. 46, 3446–3457. 10.1093/NAR/GKY088 29447383 PMC5909444

[B261] Yang Y.-G. Cortes U. Patnaik S. Jasin M. Wang Z.-Q. (2004). Ablation of PARP-1 Does Not Interfere with the Repair of DNA Double-Strand Breaks, but Compromises the Reactivation of Stalled Replication forks. Oncogene 23, 3872–3882. 10.1038/sj.onc.1207491 15021907

[B262] Yilmaz D. Furst A. Meaburn K. Lezaja A. Wen Y. Altmeyer M. (2021). Activation of Homologous Recombination in G1 Preserves Centromeric Integrity. Nature 600, 748–753. 10.1038/S41586-021-04200-Z 34853474

[B263] Yu J. Qin B. Lou Z. (2020). Ubiquitin and Ubiquitin-like Molecules in DNA Double Strand Break Repair. Cell Biosci 10, 1–10. 10.1186/s13578-020-0380-1 32071713 PMC7014694

[B264] Zahn K. E. Jensen R. B. Wood R. D. Doublié S. (2021). Human DNA Polymerase θ Harbors DNA End-Trimming Activity Critical for DNA Repair. Mol. Cel 81, 1534–1547. e4. 10.1016/J.MOLCEL.2021.01.021 PMC8231307 33577776

[B265] Zhao B. Rothenberg E. Ramsden D. A. Lieber M. R. (2020). The Molecular Basis and Disease Relevance of Non-homologous DNA End Joining. Nat. Rev. Mol. Cel Biol. 21, 765–781. 10.1038/s41580-020-00297-8 PMC8063501 33077885

[B266] Ziv Y. Bar-Shira A. Pecker I. Russell P. Jorgensen T. J. Tsarfati I. (1997). Recombinant ATM Protein Complements the Cellular A-T Phenotype. Oncogene 15, 159–167. 10.1038/sj.onc.1201319 9244351

[B267] Zong D. Callén E. Pegoraro G. Lukas C. Lukas J. Nussenzweig A. (2015). Ectopic Expression of RNF168 and 53BP1 Increases Mutagenic but Not Physiological Non-homologous End Joining. Nucleic Acids Res. 43, 4950–4961. 10.1093/nar/gkv336 25916843 PMC4446425

